# Multiple LacI-mediated loops revealed by Bayesian statistics and tethered particle motion

**DOI:** 10.1093/nar/gku563

**Published:** 2014-08-12

**Authors:** Stephanie Johnson, Jan-Willem van de Meent, Rob Phillips, Chris H. Wiggins, Martin Lindén

**Affiliations:** 1 Department of Biochemistry and Molecular Biophysics, California Institute of Technology, 1200 E. California Blvd., Pasadena, California 91125; 2 Department of Statistics, Columbia University, 1255 Amsterdam Avenue MC 4690, New York, New York 10027; 3 Departments of Applied Physics and Biology, California Institute of Technology, 1200 E. California Blvd., Pasadena, California 91125; 4 Department of Applied Physics and Applied Mathematics, Columbia University, 200 S.W. Mudd, 500 W. 120th St. MC 4701, New York, New York 10027; 5 Center for Biomembrane Research, Department of Biochemistry and Biophysics, Stockholm University, Svante Arrhenius väg 16C, SE-106 91 Stockholm, Sweden; 6 Department of Cell and Molecular Biology, Uppsala University, Box 256, SE-751 05 Uppsala, Sweden

## Abstract

The bacterial transcription factor LacI loops DNA by binding to two separate locations on the DNA simultaneously. Despite being one of the best-studied model systems for transcriptional regulation, the number and conformations of loop structures accessible to LacI remain unclear, though the importance of multiple coexisting loops has been implicated in interactions between LacI and other cellular regulators of gene expression. To probe this issue, we have developed a new analysis method for tethered particle motion, a versatile and commonly used *in vitro* single-molecule technique. Our method, vbTPM, performs variational Bayesian inference in hidden Markov models. It learns the number of distinct states (i.e. DNA–protein conformations) directly from tethered particle motion data with better resolution than existing methods, while easily correcting for common experimental artifacts. Studying short (roughly 100 bp) LacI-mediated loops, we provide evidence for three distinct loop structures, more than previously reported in single-molecule studies. Moreover, our results confirm that changes in LacI conformation and DNA-binding topology both contribute to the repertoire of LacI-mediated loops formed *in vitro*, and provide qualitatively new input for models of looping and transcriptional regulation. We expect vbTPM to be broadly useful for probing complex protein–nucleic acid interactions.

## INTRODUCTION

Severe DNA deformations are ubiquitous in biology, with a key class of such deformations involving the formation of DNA loops by proteins that bind simultaneously to two distant DNA sites. DNA looping is a common motif in gene regulation in both prokaryotes and eukaryotes (1–3). A classic example of a gene-regulatory DNA looping protein is the Lac repressor (LacI), which controls the expression of genes involved in lactose metabolism in *Escherichia coli* (1–3). LacI has two DNA-binding domains, which can bind simultaneously to two specific sites on the DNA, called operators, to form loops. Despite being one of the best-studied model systems of transcriptional regulation, the mechanics of DNA looping by LacI remain incompletely understood. One of the key outstanding issues regarding the mechanics of loop formation by LacI is that theoretical and computational modeling provide evidence for the existence of many conformations of LacI-mediated loops, but it is not clear which conformations are realized for various loop lengths, nor how many of these different conformations are relevant for gene regulation *in vivo* (4,5). Quantitative studies of looping and transcriptional regulation would be greatly aided by a better understanding of the structures of LacI-mediated loops, as many models of looping are sensitive to assumptions about the conformation of the protein and/or the DNA in the loop (5–8). Moreover, inducer molecules and architectural proteins, which are important influencers of gene regulation *in vivo*, appear to be able to manipulate these parameters (8–12). In this work we argue that at least three distinct loop structures contribute to LacI-mediated looping *in vitro* for a given DNA construct when the loop length is short (on the order of the DNA persistence length), one more than the two structures that are usually reported (13–18).

**Figure 1.**
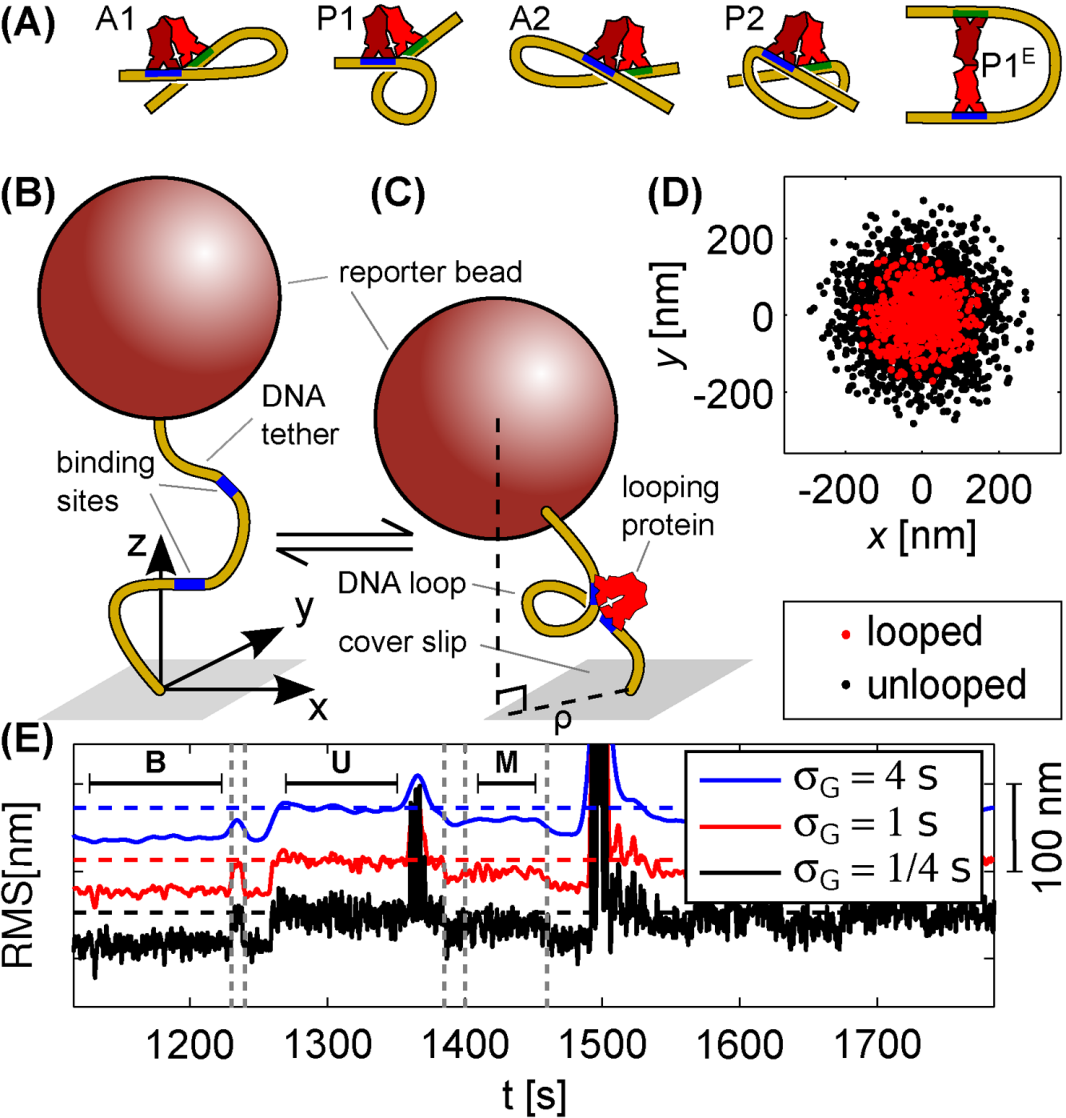
(**A**) Examples of possible LacI-mediated loops, using the notation of (6). (**B** and **C**) Tethered particle motion (TPM) setup, in which a reporter bead tethered to a cover slip by a DNA molecule is tracked as it diffuses around the tethering point. The formation of a DNA loop shortens the DNA ‘leash’, which narrows the distribution of bead positions (**D**). The degree of restriction depends not only on the length of the loop but also on the relative distance and orientation of the in- and outgoing strands, so that different loop shapes can be distinguished. (**E**) Root-mean-squared (RMS) signals, time-averaged with Gaussian filters of different kernel width σ_*G*_ (see Materials and Methods), for an example trace with an unlooped and two looped states (one long stretch of each indicated by U, M and B, respectively). Horizontal dashed lines indicate the unlooped state, offset for clarity, and vertical ones indicate potential loop–loop interconversion events.

The naturally occurring *lac* operon has three operators with different affinities for LacI (1), allowing loop formation between three different pair-wise combinations of binding sites. Most studies of looping mechanics avoid this complexity by using synthetic constructs with only two operators, but multiple loop conformations are possible even in these simplified systems. The DNA-binding domains of LacI are symmetric (19), so each operator can bind in one of two orientations, enabling four distinct loop topologies (Figure 1A). Moreover, loops could form with the LacI protein on the inside or outside of the DNA loop (5,13). In addition, it has been shown that LacI has a flexible joint, allowing the V-like shape seen in the crystal structure to adopt extended conformations as well, as in the rightmost schematic in Figure 1A (13,14,20–23). Finally, the DNA-binding domains seem to rotate easily in molecular dynamics simulations (24), which would help LacI to relax strain in the DNA of the loop (5,6,8).

Different predicted loop conformations are usually classified as differing in DNA-binding topology or in LacI conformation, with a key distinction between the two being that structures differing in DNA topology cannot directly interconvert without LacI dissociating from one or both operators, in contrast to those differing in LacI conformation (e.g. V-shaped versus extended shapes), which should be able to directly interconvert (see, for example, (13)).

The existence of multiple loop conformations for LacI-mediated loops *in vitro* has been confirmed experimentally, but identifying these experimentally observed loops with particular molecular structures is challenging. One of the most widely used techniques for studying LacI loop conformations is a non-fluorescent single-molecule technique called tethered particle motion (TPM (25); see Figure 1B), which uses the Brownian motion of a microscopic bead tethered to the end of a linear DNA to report on looping (26). TPM has resolved two looped states with a variety of synthetic and naturally occuring DNA sequences (13–17,27). However, the structural basis of these two states is currently a subject of debate. Importantly, direct interconversions between the two looped states have been observed in TPM experiments with 138 bp and 285 bp loops. This strongly suggests that a conformational change of LacI occurs in these loops, presumably a transition between a V-like and a more extended state (13,14), since a change of loop topology would require an unlooped intermediate.

There is also evidence from both ensemble and single-molecule fluorescence resonance energy transfer (FRET) experiments with synthetic, pre-bent loop sequences, whose conformations can be determined computationally, for at least two (22,28) and possibly three (23) coexisting loops differing in both DNA topology and LacI conformation. However, it is as yet unclear which of the structures observed by FRET correspond to the states observed by TPM, and whether three loop conformations might also coexist in the loops formed from generic rather than pre-bent DNA sequences.

One difficulty in determining the number of looping conformations in TPM measurements is that not all loop conformations produce distinct TPM signals (7,18), raising the possibility that the actual number of conformations might be greater than two. Indeed, elastic modeling consistently predicts the coexistence of more than two conformations for a single looping construct, either through direct arguments (i.e. finding multiple loop structures with comparable free energies (7,8)) or indirectly by predicting that the most stable V-shaped loops and the most stable extended loops have different DNA topologies (5,6). In the latter case, the lowest energy states of the V-shaped and the extended conformations would be geometrically unable to interconvert directly with each other, since they differ in DNA topology. Thus, previous reports of direct loop–loop interconversions (13,14) would have to be explained by the existence of at least one additional loop structure that shares a DNA topology with one of the lowest energy states.

These considerations suggest two questions to address in order to make progress toward identifying the loop structures relevant for looping *in vitro*: (1) is there evidence for more than two loop structures underlying previously reported TPM data, as would be expected from elastic modeling and from FRET results with pre-bent sequences? and (2) which of the observed states interconvert directly, identifying them as differing in LacI conformation rather than DNA-binding topology?

Shorter loop lengths (i.e. shorter than the persistence length of DNA, roughly 150 bp) tend to enhance the free energy differences between loop structures, and so provide an interesting opportunity to look for detectable signatures of additional loop structures, and to determine which state(s) directly interconvert. We recently reported TPM data of two apparent looped states for loop lengths around 100 bp (16), but the presence or absence of direct interconversions between the two states was not addressed. Here, we revisit these data to address the questions of direct interconversions and the number of looped states more rigorously. We provide evidence for the presence of a third looped state in addition to the two previously reported, and we demonstrate direct interconversions between two of the three states.

Detection of direct loop–loop interconversions requires a high time resolution, which is especially difficult to obtain at short loop lengths where the signal-to-noise ratio of TPM data is comparatively small. To meet this challenge, we have developed a powerful set of analysis techniques for TPM data, based on inference in hidden Markov models (HMMs (29)) using variational Bayesian (VB) methods (30–35). HMMs are widely used to analyze ion channel (36), optical trapping (37), magnetic tweezers (38), single-molecule FRET (31,32,34,35,39) and single-particle tracking (33) experiments. Our toolbox, which we call vbTPM, offers several advantages over existing TPM analysis techniques, including improved resolution, an objective criterion to determine the number of (distinguishable) DNA/protein conformational states, robustness against common experimental artifacts and a systematic way to pool information from many trajectories despite considerable cross-sample heterogeneity.

vbTPM should benefit a broad community of users, as TPM is a versatile and widely used single-molecule technique, with its simplicity, stability, ability to measure DNA–protein interactions at very low applied tension (40,41), and potential for high throughput (42) making it an attractive tool for *in vitro* studies of protein–nucleic acid interactions that loop or otherwise deform DNA (15–18,25,26,43–52). Moreover, our results from applying vbTPM to TPM data on short DNA loops provide important new inputs for a comprehensive understanding of LacI-mediated DNA looping *in vitro* and quantitative models of transcriptional regulation *in vivo*.

## MATERIALS AND METHODS

### TPM data

We present new analysis of previously published data (16) for constructs that contain 100–109 bp of either a synthetic random sequence called E8 (53,54) or a synthetic, strong nucleosome positioning sequence called 601TA (abbreviated TA) (53–55) in the loop, flanked by the strongest naturally occurring LacI operator O1 and an even stronger synthetic operator called Oid. We denote these constructs E8*x* and TA*x*, where *x*=100–109 and refers to the length of the loop, excluding the operators. The O1 and Oid operators are 21 and 20 bp long, so the distance between operator centers is thus *x*+20.5 bp. For ease of comparison between our results and others’, we use loop length, not distance between operator centers, when quoting other's results. The *in vitro* affinities of LacI for the O1 and Oid operators are roughly 40 and 10 pM, respectively (16,56–59). The total lengths of the DNA tethers range from 458 to 467 bp, depending on the length of the loop (16).

For every tethered DNA, we collected 10 min of calibration data in the absence of LacI, followed by roughly 20–100 min of looping data in the presence of 100 pM LacI, purified in-house. Data sets for each loop length typically contain 50–100 TPM trajectories. We used a standard bright-field microscopy-based TPM setup, where 490 nm diameter polystyrene beads are tracked in the *xy*-plane with video microscopy at 30 Hz, and the resulting trajectories then drift-corrected using a first-order Butterworth filter with a 0.05 Hz cutoff frequency (see (16) for detailed experimental and analysis procedures). As noted below, this drift-corrected data was used as the input for the HMM analysis (and not the subsequently Gaussian-filtered root-mean-square (RMS) trajectories that are described in (16)).

In addition to the pre-existing data, we also obtained calibration trajectories from constructs with total lengths 450 bp (‘E894’ of (16)), 735 bp (‘wild-type’ of (60)) and 901 bp (‘PUC306’ of (15,60)). Data for these constructs were obtained in the absence of LacI only.

### RMS analysis

The RMS trace of a tether is the square root of a running average of the variance of the bead's position, $\sqrt{\left\langle \rho ^2\right\rangle }$. We followed the procedures of (16), in which *ρ* was calculated from drift-corrected *x* and *y* bead positions, as described in the previous section, and then convolved with a Gaussian filter, except here we varied the standard deviation σ_*G*_ of the Gaussian filter kernel for the running average, rather than keeping it fixed at 4 s as in (16). To count the number of states, we determine the number of peaks in RMS histograms by eye.

### Diffusive HMM for single trajectories

vbTPM uses a diffusive HMM to describe the bead motion and looping kinetics in a manner that directly models bead positions instead of RMS traces. In an HMM, kinetics are modeled by a discrete Markov process *s*_*t*_, *t* = 1, 2, …, *T*, with *N* states (e.g. *s*_*t*_ = 1 when unlooped, *s*_*t*_ = 2 when looped, etc.), a transition probability matrix $\boldsymbol {A}$ and an initial state distribution $\boldsymbol{\pi }$,

(1) \begin{equation*} p(s_t|s_{t-1},\boldsymbol {A})=A_{s_{t-1}s_t},\quad p(s_1|\boldsymbol{\pi })=\pi _{s_1}. \end{equation*}

The physics specific to TPM are contained in the emission model, which describes the motion of the bead for each hidden state. We use a discrete-time model of overdamped 2D diffusion in a harmonic potential that has been suggested as a simplified model for TPM (61,62). This means that the probability distribution of each bead position is Gaussian and depends conditionally on the hidden state and previous position,

(2) \begin{equation*} p(\boldsymbol{x}_t|\boldsymbol{x}_{t-1},s_t,\boldsymbol{K},\boldsymbol{B}) =\frac{B_{s_t}}{\pi } e^{-B_{s_t}(\boldsymbol{x}_t-K_{s_t}\boldsymbol{x}_{t-1})^2}. \end{equation*}

The emission parameters *K*_*j*_ and *B*_*j*_ are related to the spring and diffusion constants of the corresponding hidden states. More insight into their physical meaning can be gained by noting that with a single hidden state, Equation (2) describes a Gaussian process with zero mean and

(3) \begin{eqnarray*} &{\rm RMS}= \sqrt{\left\langle \rho ^2\right\rangle }=\sqrt{\left\langle \boldsymbol{x}^2\right\rangle }=(B(1-K^2))^{-1/2},\nonumber \\ &\left\langle \boldsymbol{x}_{t+m}\cdot \boldsymbol{x}_t\right\rangle /\langle \boldsymbol{x}^2\rangle =K^{|m|}\equiv e^{-|m|\Delta t/\tau }, \end{eqnarray*}

where Δ*t* is the sampling time, and $\tau =-\frac{\Delta t}{\ln K}$ is a bead correlation time (see Section S2 in the Supplementary Information (SI)). This model captures the diffusive character of the bead motion while retaining enough simplicity to allow efficient statistical analysis.

### Inference and model selection

To analyze TPM trajectories using the above model, we apply a VB technique (30) that has previously been used in the analysis of other single-molecule data (31–35), but has not been applied to TPM data so far. VB methods can determine both the most likely number of hidden states *N* and the most likely parameters $\theta = \lbrace \boldsymbol {A}, \boldsymbol{\pi }, \boldsymbol{K}, \boldsymbol{B}\rbrace$ for the model. Models with more states and parameters can generally model the data more closely, but may overfit the data by attributing noise fluctuations to separate states. VB methods perform model selection by ranking models according to a lower bound *F*_*N*_ on the log evidence ln *L*_*N*_. The evidence *L*_*N*_ is the marginal probability of observing the measurement data, obtained by integrating out all model parameters *θ* and hidden state sequences {*s*_*t*_} from the joint probability $p(\lbrace \boldsymbol{x}_t\rbrace , \lbrace s_t\rbrace , \theta \,|\, N)$,

(4) \begin{equation*} F_N \lesssim \ln L_N=\ln \sum _{s_1,s_2,\ldots }\int p(\lbrace \boldsymbol{x}_t\rbrace ,\lbrace s_t\rbrace |\theta ,N)p(\theta |N) d\theta .\end{equation*}

The model with the highest lower bound log evidence *F*_*N*_ can be interpreted as the model that exhibits the best ‘average’ agreement with the data over a range of parameters, thereby eliminating models that overfit the data and only show good agreement for a narrow parameter range. VB analysis requires us to parameterize our prior knowledge (or ignorance) about parameter values in terms of prior distributions *p* (*θ *| *N*). We choose ‘uninformative’ priors to minimize statistical bias. VB analysis also yields parameter information in terms of (approximate) posterior distributions on *θ*, which are optimized numerically to maximize *F*_*N*_ when fitting a model to data. We generally report parameter values as expectation values of these distributions. Further details are given in the SI and software documentation (see below).

### Downsampling

To decrease the computational cost associated with analysis of large data sets, we downsample by restricting the hidden state changes to occur on multiples of *n* data points. By downsampling only the hidden states, and not the TPM data, we avoid discarding valuable information about bead relaxation dynamics (62,63). We use *n* = 3 except where noted otherwise. With an original sampling frequency of 30 Hz and *K* ≳ 0.4 (τ ≳ 1/30 s) in our data (see Results), the shortest possible state lifetime (1/10 s after downsampling) is thus at most three times larger than the bead correlation time.

### Synthetic data

We generate synthetic data by direct simulation of Equations (1) and (2), followed by application of a first-order Butterworth filter with 0.05 Hz cutoff frequency to simulate drift-correction (15,16). To generate reasonable parameter pairs, we use the empirical fit $\tau =0.018 {\rm RMS}-0.079$, with *τ* in seconds and RMS in nm, and then compute *K*, *B* from Equation (3). For analysis, we use the same settings (priors, etc.) as for real data.

### Pooled analysis of multiple trajectories

To make full use of the high-throughput capabilities of TPM, it is advantageous to pool information from many trajectories in a systematic way. Indeed, we will see below that this is necessary to unambiguously resolve direct interconversions between looped states. Two problems must be solved in order to pool information from multiple trajectories. First, TPM data contain artifacts, e.g. transient sticking events or tracking errors (described in more detail below). Such spurious events are specific to each trajectory and should not be pooled. Second, variations in bead size, attachment chemistry, etc., create significant variability between beads in nominally equal conditions (e.g. DNA construct length and LacI concentration (16)), making it infeasible to fit a single model to multiple trajectories even without spurious events.

To address the first problem, we extend the single trajectory HMM with a second type of hidden state, *c*_*t*_, such that *c*_*t*_ = 1 indicates genuine looping dynamics governed by the simple model described above. When *c*_*t*_ > 1, the bead motion is instead assumed to arise from some kind of measurement artifact, which is modeled by a different set of emission parameters $\hat{B}_{c_t},\hat{K}_{c_t}$. We assume the genuine states, *s*_*t*_, to evolve independently of spurious events. Similarly, spurious events *c*_*t*_ > 1 can interconvert independently of the underlying genuine state, but transitions out of *c*_*t*_ = 1 depend on *s*_*t*_, to allow for possibilities such as transient sticking events being more frequent in a looped state when the bead is on average closer to the cover slip. These assumptions mean that the joint transition probability of *s*_*t*_, *c*_*t*_ factorizes as

(5) \begin{equation*} p(s_{t+1},c_{t+1}|s_t,c_t)=p(s_{t+1}|s_t)p(c_{t+1}|s_t,c_t). \end{equation*}

We therefore refer to it as a (variant of a) factorial HMM (64). As before, $p(s_{t+1}|s_t)=A_{s_ts_{t+1}}$, but transitions involving the spurious states are described by two new transition matrices $\hat{\boldsymbol {A}}$ and $\hat{\boldsymbol {R}}$,

(6) \begin{equation*} p(c_{t+1}|s_t,c_t) =\left\lbrace \begin{array}{ll}\skew9\hat{A}_{s_tc_{t+1}},&\text{ if } c_t=1,\\ \hat{R}_{c_tc_{t+1}},&\text{ if } c_t {>} 1.\\ \end{array} \right. \end{equation*}

To deal with bead-to-bead variability, we adopt an empirical Bayes (EB) approach that derives from a recently developed analysis technique for single-molecule FRET data (34,35). In EB analysis, the prior is interpreted as the distribution of model parameters across the set of trajectories and is learned from the data to maximize the total lower bound log evidence. In this manner, similarities between trajectories are exploited to obtain more accurate parameter estimates. We restrict EB analysis to transition probabilities and emission parameters of the genuine states (*s*_*t*_ in Equations 5–6), while priors describing spurious states are held fixed.

Pooled analysis using EB and the factorial model is performed in four steps, summarized in Section S1. First, we perform VB analysis, learning the optimal number of states for each trajectory. Second, genuine and spurious states are classified using an automated procedure (see Equation (7) below), and verified manually using a graphical tool. In practice, very few corrections to the automated classification are needed. Third, factorial models are generated by translating the spurious states of the simple HMMs into *c*_*t*_ > 1-states (Equations 5–6), and reconverged using a VB algorithm. Finally, these factorial models are used as an initial guess for the EB algorithm. Since EB analysis requires all models to have the same number of genuine states, some factorial models also have to be extended with extra unoccupied states. Information can then be extracted from the optimized prior distributions. Further details are given in the software documentation.

### Implementation

vbTPM runs on Matlab with inner loops written in C and includes a graphical tool for manual state classification. Source code and software documentation are available at http://vbtpm.sourceforge.net.

## RESULTS

### Improved resolution on synthetic data

A simple and common way to analyze TPM data is in terms of RMS values, which are the square root of the bead position variance, or the projected distance ρ between the bead center and tether point (Figure 1E). Transitions can be extracted by thresholding RMS traces, and the number of states by counting peaks in RMS histograms (13,16,17,26,45,65,66). However, the RMS signal must be smoothed in order for the transitions to appear. This degrades the time resolution (67), and a direct analysis of bead position traces, such as vbTPM, would likely do better in this respect (68). As noted above, this is of particular interest when determining whether or not apparent loop–loop interconversions are in fact separated by short unlooped intermediates.

We have tested vbTPM on synthetic data and compared its ability to resolve close-lying states with that of the RMS histogram method. Two states can be difficult to resolve either due to similar RMS values or short lifetimes. Our state detection tests (see Supplementary Figure S2–S4) show that vbTPM offers a great improvement over RMS histograms in the latter case, which is precisely the case that matters most for the question of direct interconversions that we address here. For example, two states separated by 40 nm are resolved by vbTPM at a mean lifetime of about 0.5 s, while lifetimes of 4–8 s are necessary for states to be resolvable in RMS histograms (Supplementary Figure S2). This order of magnitude improvement mainly reflects the detrimental effects of the low-pass filter used in the RMS analysis (see RMS analysis in Materials and Methods). The difference diminishes for more long-lived states, and with a mean lifetime of 30 s, the spatial resolution is about 15 nm for both methods (Supplementary Figures S3 and S5).

Our tests with synthetic data further show that the parameters, including transition rates, are faithfully recovered by vbTPM, and that all of these results are insensitive to downsampling by the factor of three that we use when analyzing real data (Supplementary Figures S5–S7).

### Detection of experimental artifacts

A striking illustration of the improved time resolution of vbTPM is the ability to detect and classify short-lived experimental artifacts in the data. Our normal TPM protocol starts with a short calibration run in the absence of the looping protein for quality control reasons (16). Here, we expect only one state, that of the fully extended tether. However, analyzing calibration data for three different construct lengths, we find more than one state in most trajectories, although a single state usually accounts for most (∼99%) of the trajectory.

**Figure 2.**
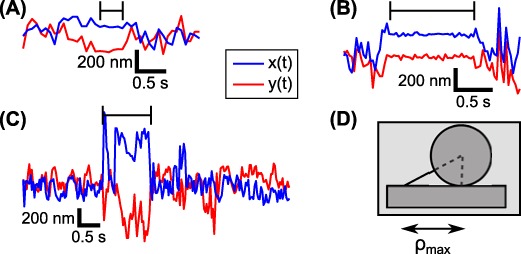
Examples of spurious events in calibration data (i.e. in the absence of repressor). Spurious events are indicated by horizontal markers above the red and blue traces of the bead's x and y positions. (**A**) and (**B**) show ‘sticking events’ (non-specific, transient attachments of the bead to the surface, the DNA to the bead, etc.), while (**C**) contains an excursion larger than the physically possible maximum, $\rho _\text{max}$, as shown in (**D**). This could be due to a tracking error, for example when an untethered bead diffuses through the field of view. Note that the events shown here are all on the second timescale, and hence undetectable with the temporal resolution of about 11 s in our previous RMS-based analysis (16).

**Figure 3.**
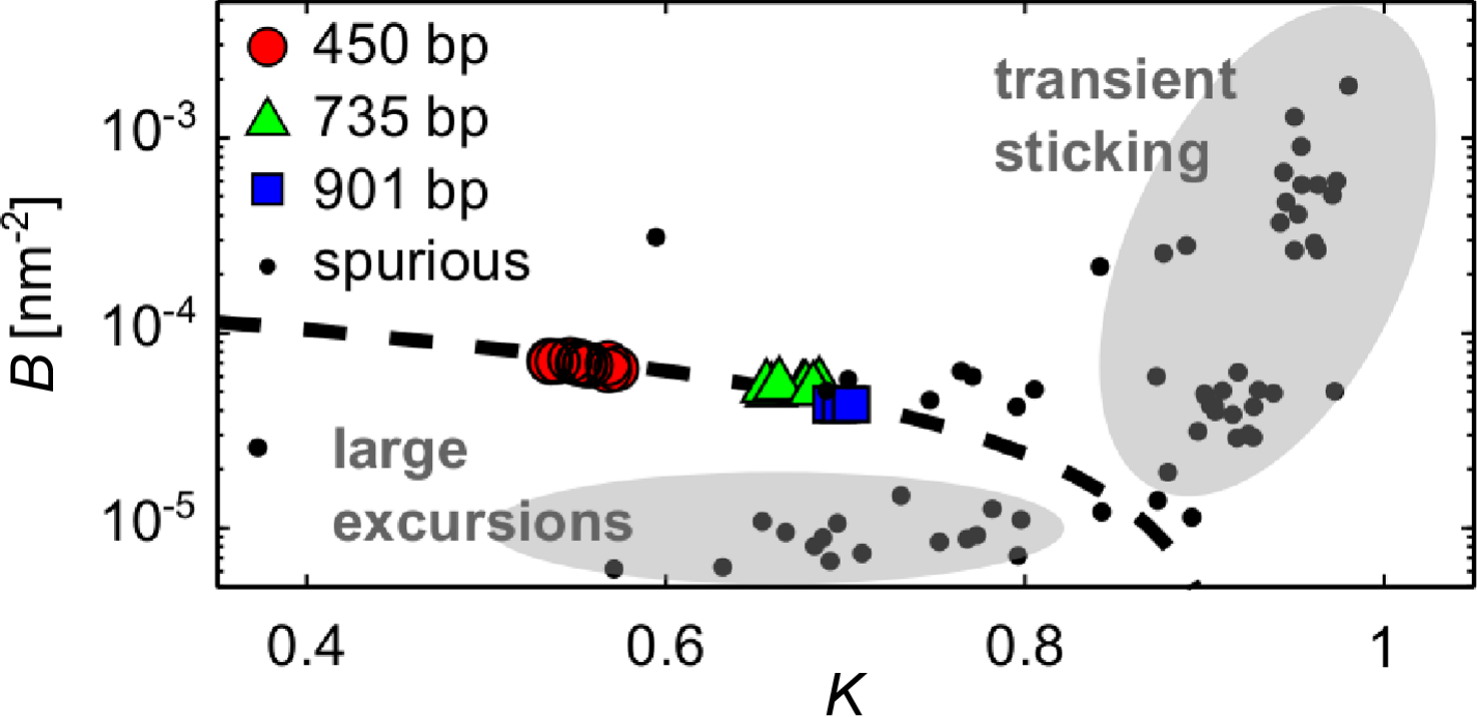
Scatter plot in the (*K*, *B*) plane of genuine and spurious states in trajectories without LacI from three different tether lengths. The genuine states, colored according to tether length, are defined as the most long-lived state in each trajectory, and fall close to the empirical fit *B* = (1.84 − 2*K*) × 10^−4^ nm^−2^ (dashed line, note log-scale on the *B*-axis). Spurious states (dots) scatter off of this line. Gray ellipsoids indicate rough parameter trends for sticking and tracking errors (large excursions), respectively.

**Figure 4.**
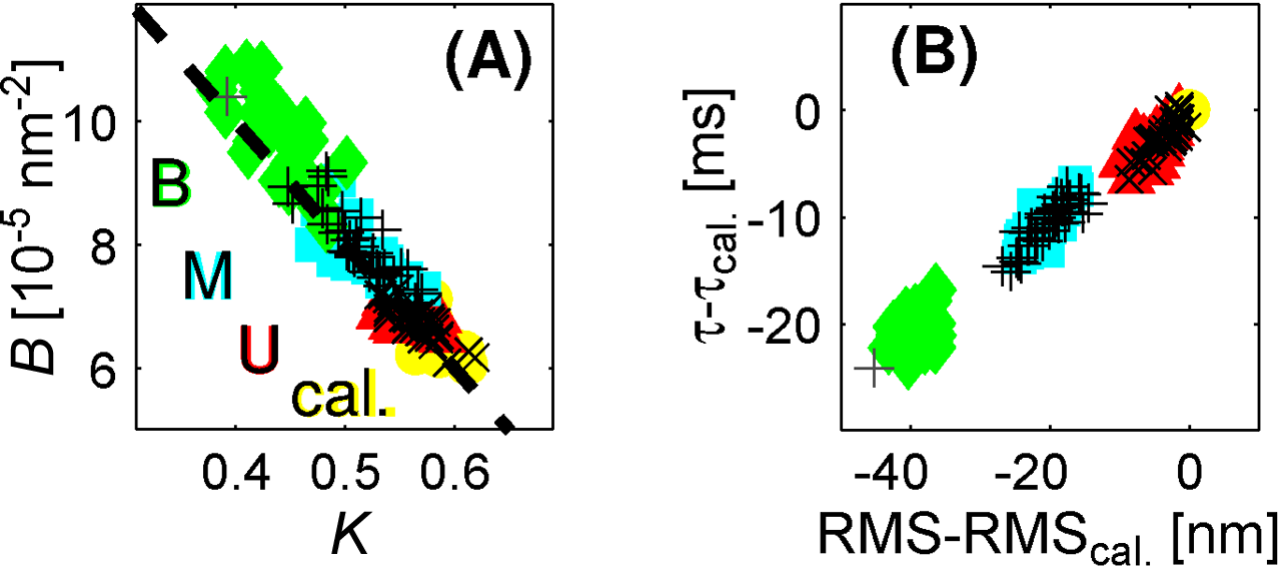
Clustering of LacI-induced looped and unlooped genuine states in the E8106 construct. States U, M and B in three-state trajectories are represented as filled symbols, while states in two-state trajectories are plotted as +'s (for the looped state) and x's (for the unlooped state). (**A**) Raw emission parameters *K*, *B*. The dashed line is the linear fit from Figure 3. (**B**) Same states as in A, but plotted as RMS values and relaxation times τ (see Equation (3)) relative to the calibration (that is, no-LacI) states for each tether. From now on, we will plot states in these more intuitive and homogeneous terms.

Inspection of the coordinate traces (that is, the *x* and *y* positions of the bead as functions of time) reveals the dominant state to correspond to normal, ‘genuine’ bead motion, while the extra ‘spurious’ states are associated with obvious irregularities in the data. Many of these are too short-lived to show up in the time-averaged RMS traces. Almost all can be interpreted as either transient sticking events (Figure 2A and B), where the motion in *x* and *y* simultaneously and abruptly goes down dramatically, or brief excursions beyond the limit set by the tether length (Figure 2C and D), caused by breakdowns of the tracking algorithm when, for example, free beads diffuse through the field of view. Some spurious events are described as more than one state in the vbTPM analysis. A scatter plot of the emission parameters *K* and *B* for detected states (see Equations (2,3)) shows different patterns for genuine and spurious states (Figure 3). Genuine states fall along a curve in the *K*, *B* plane, while the spurious states scatter. This makes physical sense, since the genuine dynamics are governed by a single parameter, the effective tether length, while the spurious states are of diverse origins. This pattern persists also in trajectories with looping, with the genuine looped states continuing along the curve indicated by the calibration states (Figure 4A).

The *K*, *B* values of different trajectories vary significantly, but it turns out that within fitting uncertainty, most states of individual trajectories satisfy

(7) \begin{equation*} K_{{\rm gen.}}\le K_{\rm cal.}\text{, and }B_{{\rm gen.}}\ge B_{\rm cal.}, \end{equation*}

with (·)_cal_*_._* and (·)_gen._ denoting genuine state parameters of calibration and looping trajectories, respectively. Most spurious states violate at least one of these inequalities. An intuitive rationale for this rule is that *K* (*B*) tends to decrease (increase) with decreasing tether length as seen in Figure 3. Looping decreases the effective tether length, as does the slight bending of the operator sites upon LacI binding (16,19).

The upshot of the different behaviors of genuine and spurious states shown in Figures 3 and 4A is that Equation (7), plus an additional lower threshold on RMS values (see Equation (3)) to catch sticking events near the tethering point, can be used to computationally label genuine versus spurious states. Very few exceptions remain to be corrected manually. While spurious states make the HMM analysis more complicated, they constitute a sufficiently minor fraction of most trajectories, such that their presence does not significantly affect the average looping properties (see Supplementary Figures S8 and S9), and hence their presence does not invalidate previous TPM results that did not remove them.

### More than two looped states

We used vbTPM to examine looping at 100 pM LacI in E8*x* and TA*x* constructs, where ‘*x*’ indicates the loop length, ranging from 100 to 109 bp (16), and E8 and TA are two different DNA sequences in the loop (see Materials and Methods). We applied Equation (7) complemented by visual inspection to identify genuine states, and from now on, we will understand all ‘states’ to be genuine unless stated otherwise. Most trajectories exhibit one to three states in the presence of LacI.

We discard trajectories with only one state, as a complete lack of looping activity might reflect defective constructs, surface attachment, or LacI molecules (16). We also discard a small number of trajectories with four states, where inspection reveals either a state split by bursts of spurious events (resulting in artificial differences in state lifetimes) or a genuine-looking state with very low RMS that can be attributed to a sticking event near the tethering point. Thus, our HMM analysis is at first glance consistent with earlier findings of two distinguishable looped states in these constructs (16). We denote the states from trajectories with three states ‘unlooped’ (U), ‘middle’ (M) and ‘bottom’ (B), in keeping with the conventions of (16,17), in which ‘middle’ and ‘bottom’ refer to the tether lengths of the two distinguishable looped states relative to the unlooped state.

We find, however, that not all of the remaining trajectories in a population show all three states; some have only one of the two looped states. The two- versus three-state trajectories display a striking pattern that we will introduce using the E8106 construct. As shown in Figure 4A, a scatter plot of the emission parameters for three-state trajectories (colored symbols) produces partly overlapping clusters in the *K*, *B*-plane, corresponding to the three observed states (U, M, B). Some contributions to the parameter noise, such as bead size variations, might be correlated between states, and can thus be reduced by normalization. Indeed, visualizing the states relative to their calibration states (Figure 4B) produces well-separated state clusters. These clusters allow us to classify the states in the trajectories with only two states (+ and x in Figure 4), by comparison to the clusters formed by the three-state trajectories. In 37 out of 38 two-state trajectories, the two states coincide with the U and M states. That is, in trajectories that only exhibit one of the two looped states, for the E8106 construct that looped state is *always* the ‘middle’ state.

One possible explanation for this pattern is that it results from insufficiently equilibrated three-state kinetics—that is, all two-state trajectories are really three-state trajectories that were not observed long enough. In Section S6, we present simulated data to show that under this null hypothesis we would expect significantly more three-state trajectories than we actually observe in most constructs. In other words, the number of two-state trajectories found in our analysis is not consistent with a simple equilibration effect. We hypothesize instead that there are two underlying populations in our data, one population that has two states (one looped and one unlooped), and one population with three states.

**Figure 5.**
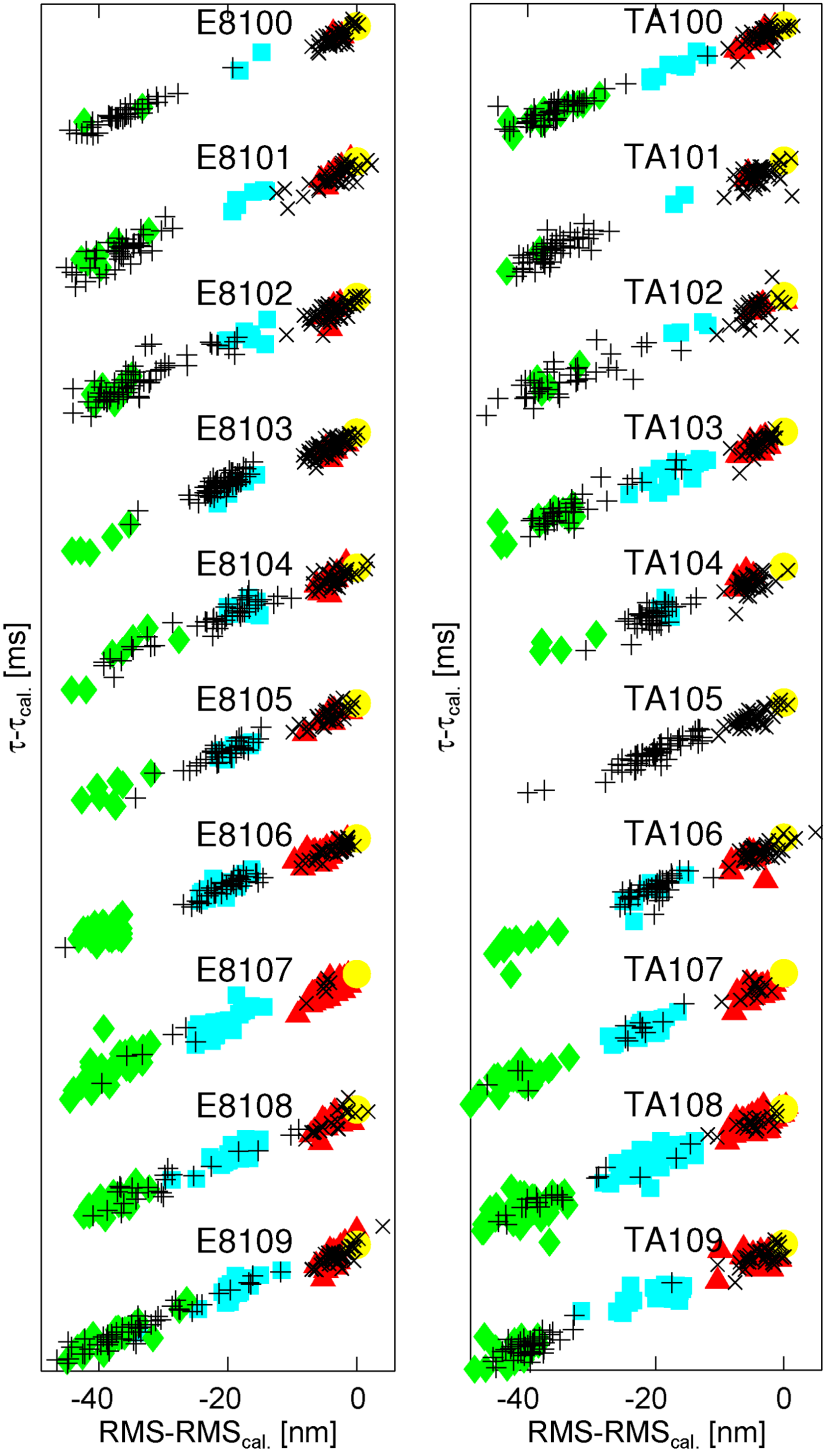
Clustering of looped and unlooped states for E8*x* and TA*x* constructs, with loop lengths *x* in the range 100–109 bp. The states are colored and aligned as in Figure 4B, and offset in the τ direction for clarity.

Similarly, we find that a sub-population of LacI that is somehow unable to support the B state is also unlikely, as different cluster patterns appear with other loop lengths and sequences. As shown in Figure 5, when we subject E8 and TA constructs spanning one helical repeat to the same analysis, we see some constructs (e.g. E8103, TA104, E8105, TA106) mimic the 2+3-state pattern of E8106, but in others (E8100-101, TA100-101, TA109) the looped state in two-state trajectories is the B rather than M state. Moreover, while there is also one case for each sequence with almost exclusively three-state (E8107) or two-state (TA105) trajectories, the identity of the looped state in two-state trajectories exhibits a clear phasing that correlates with loop length, and therefore with the helical repeat of the DNA. In particular, when the operators are in-phase and looping is maximal, demonstrated in our previous work to occur around 106 bp (16), the looped state in two-state trajectories is predominately the M state, whereas when the operators are out-of-phase, around 100 or 110 bp (16), two-state trajectories contain primarily the B state as the looped state.

**Figure 6.**
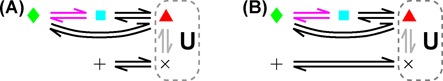
Proposed kinetic models for the ‘2+3’ pattern of states observed in Figure 5, with slow interconversions (gray arrows) between two- and three-state trajectories occurring via multiple unlooped states. Symbols and colors follow those of Figure 5. (**A**) Kinetic model for in-phase operators, e.g. around 106 bp loops, where looping is maximal and the looped state in two-state trajectories is the M state, represented by a ‘+’ as in Figure 5. (**B**) Kinetic model for out-of-phase operators, e.g. around 100 or 110 bp loops, where looping is minimal and the looped state in two-state trajectories is the B state, again represented by a ‘+’. Purple arrows represent putative direct loop–loop interconversions, whose existence is explored in the last section of the Results.

We propose a structural explanation for these observations, namely, that the M state in trajectories exhibiting only two states corresponds to a different loop structure than the M state in trajectories with three states, and that interconversion between the two- and three-state regimes occurs slowly, via multiple unlooped states, as sketched in Figure 6. A further line of evidence supporting this explanation concerns the question of whether or not the M and B states in three-state trajectories interconvert: if the M state can interconvert with the B state in three-state trajectories, but the M state in two-state trajectories never interconverts with the B state (because these trajectories show no B state), then it is likely that these two M states (interconverting and not interconverting) are structurally different. Moreover, as noted in the Introduction, the question of direct interconversions can provide insight into what structures might underlie the interconverting and non-interconverting M and B states: if two looped states interconvert without passing through the unlooped state, this would indicate that the involved states have the same DNA-binding topologies, since a change of binding direction would require an unlooped intermediate. To address these questions, we now ask if the looped states in three-state trajectories interconvert directly—that is, if one of the blue states in Figure 5 can be followed by a green state without passing through a red state, and similarly for green to blue.

### Direct loop–loop interconversions

Detecting direct interconversions between looped states is difficult. Potential events can be spotted in RMS traces, but as illustrated in Figure 1E, their interpretation depends on the filter width σ_*G*_, and we cannot exclude the presence of short unlooped intermediates by eye. To test whether the increased temporal resolution of our HMM-based analysis could improve upon the detection of short unlooped intermediates, we generated synthetic data using realistic parameters obtained from the E8106 and E8107 constructs with three genuine states, with spurious states removed. The transition probabilities *A*_*ij*_ from these fits allow loop–loop interconversions, typically no more than 10 per trajectory, but we also generated data without interconversions by setting *A*_*BM*_ = *A*_*MB*_ = 0.

**Figure 7.**
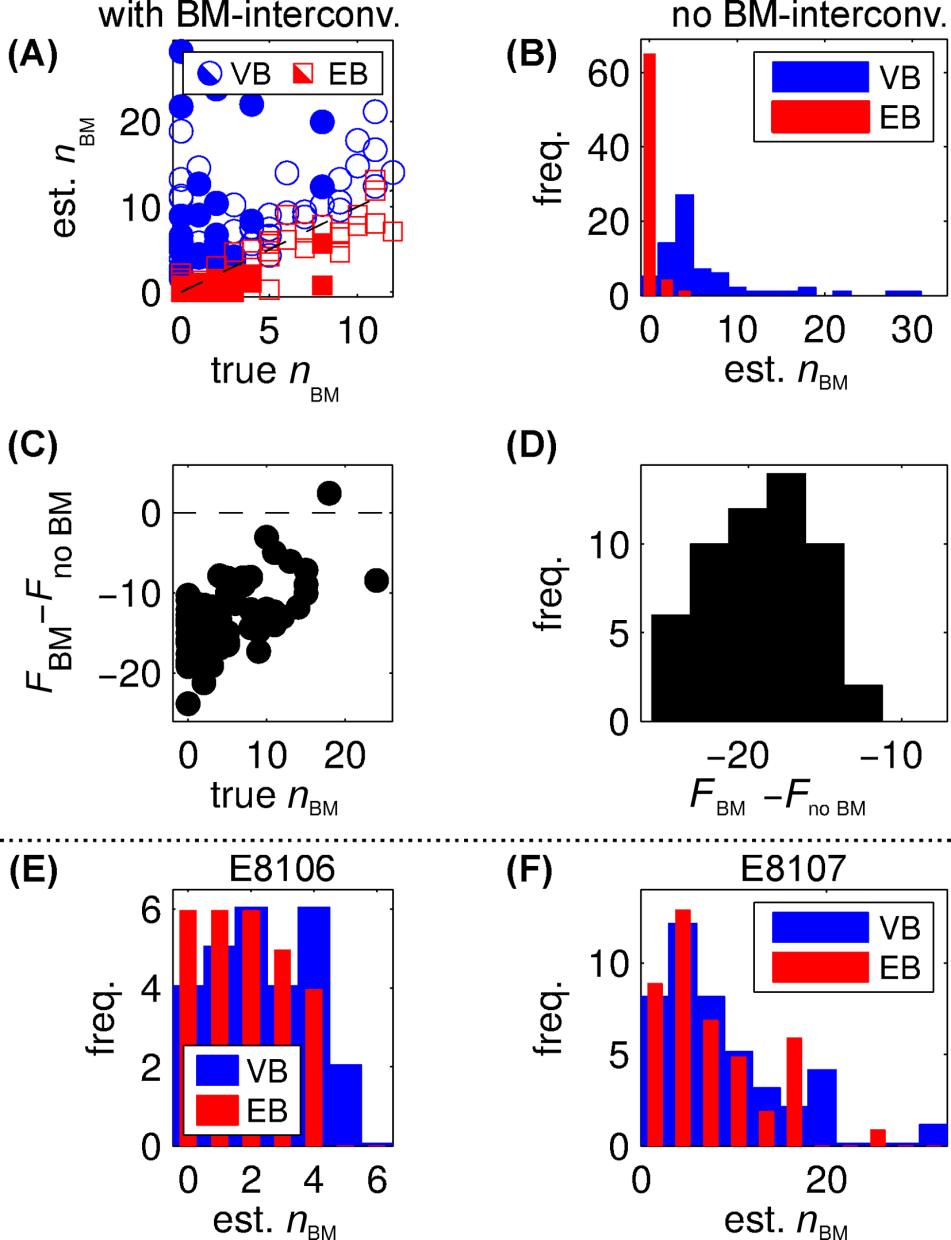
Detecting direct loop–loop interconversions. (**A** and **B**) Counting the number of $B \leftrightharpoons M$ interconversions, *n*_*BM*_, detected in synthetic data, with (A) and without (B) such transitions actually present, when trajectories are considered one at a time (‘VB’), or using an EB approach to analyze all trajectories from the same data set at once (‘EB’). The dashed black line in (A) indicates where the estimated number of interconversions equals the true number. Since most blue points lie above this line, the VB approach overestimates the number of true interconversions; but the EB analysis either accurately counts such transitions, or slightly underestimates them. Filled and open symbols in (A) refer to trajectories created from E8106 and E8107 trajectories respectively. (B) shows a histogram of the number of direct interconversions per trajectory rather than a scatter plot, because the true number of interconversions is zero; here the EB analysis accurately estimates that there are few or no interconversions, whereas the VB approach incorrectly detects direct interconversions where there are in fact none. (**C** and **D**) VB analysis of single synthetic trajectories prefers models without BM-interconversions, whether they are present (C) or not (D), probably since they are rare events. Every point and histogram count represents a single trajectory, and *F*_(…)_ is the approximate log evidence, Equation (4), for the different models. Higher *F*-values indicate better fits, so *F*_*BM*_ < *F*_*noBM*_ means that models with no interconversions are preferred by this analysis. (**E** and **F**) Analysis of real data yields a substantial number of interconversions even with the EB scheme, a strong indication that they are in fact present. That is, histograms of the number of direct interconversions per trajectory have significant weight at values above the zero bin, when estimated both by the VB approach (which tends to overestimate interconversions) and the EB approach (which accurately or slightly underestimates them). The position traces for the E8106 and E8107 constructs are included as supplementary data.

Refitting these synthetic data sets with our standard settings, we find that the HMM algorithm overcounts the number of looped state interconversions, *n*_*BM*_, even when they are absent in the data (Figure 7A and B). Moreover, models that disallow direct BM-interconversions generally get higher *F*-values (related to goodness of fit; see Equation (4)) than models that allow interconversions, even when such interconversions are actually present (Figure 7C and D). Thus, we cannot settle the question of direct loop–loop interconversions by analysis of single trajectories, probably because the number of such interconversions per trajectory is too few in our data and in the synthetic data we create from it.

To overcome these limitations, we perform pooled analysis of multiple trajectories. The difficulty in this analysis is that we cannot simply fit a single model to multiple trajectories, because of the large bead-to-bead variations in motion parameters (*K*, *B*) seen in Figure 4A, and the varying numbers of spurious states in different trajectories seen in Figure 3, which differ in both number and parameter values for each trajectory. To solve these problems, we first extend our HMM to split spurious and genuine states into two separate hidden processes (what we call a factorial HMM; see Materials and Methods). Second, we implement an EB approach (34,35) (see Materials and Methods), which optimizes the prior distributions based on the variability of genuine states in different trajectories. This allows information from the whole data set to be used in interpreting each single trajectory and has been shown to greatly improve the resolution in single-molecule FRET data (34).

Analysis of synthetic data, where the true number of interconversion events is known, shows clear improvements when using our EB analysis in comparison to normal VB methods that analyze each trajectory individually. As shown in Figure 7A, the tendency to overestimate the number of BM-interconversions is eliminated when the EB scheme is applied, and almost no such transitions are detected in trajectories where they are absent (Figure 7B). This shows that the EB scheme can reliably detect the presence of direct BM-interconversions, although it tends to undercount when transitions are very rare (see also Supplementary Figure S11).

EB analysis of experimental data shows a substantial number of direct BM-interconversions in three-state trajectories from E8106 and E8107 (Figure 7E and F), as well as from the other constructs where there are a significant number of three-state trajectories present (Supplementary Figures S12 and S13). This is a strong indication that direct loop–loop interconversions do occur in the short-loop-length regime studied here.

This evidence for direct loop–loop interconversions, taken together with the overrepresentation of two-state trajectories discussed in the previous section, leads us to hypothesize that most constructs in Figure 5 exhibit at least three distinct loop structures, one more than previously reported in a single construct by TPM (13–18): an M and a B state that can interconvert without an unlooped intermediate, suggesting that they share the same DNA topology but different LacI conformations (e.g. a V-shaped and an extended conformation); and an M (for in-phase operators) or B (for out-of-phase operators) state that cannot directly interconvert with another looped state.

## DISCUSSION

We have developed a Bayesian analysis method for TPM data based on HMMs, called vbTPM. A major advance offered by our method is improved time resolution, which stems from our direct analysis of position data, thus avoiding the time-averaging required to produce readable RMS traces (Figure 1). We are not the first to exploit this possibility. Beausang and Nelson (63) used manually curated training data to construct detailed models of the diffusive bead motion for the looped and unlooped states, and combined them with a two-state HMM to extract interconversion rates. Manzo and Finzi (68) modeled bead positions as uncorrelated zero-mean random variables, and used change-point and hierarchical clustering methods to segment TPM position traces in order to extract dwell time statistics.

Our new analysis tool improves on previous methods in several ways. Compared to the change-point method (68), we use a noise model that accounts for correlations in the bead motion, which eliminates the need to filter out short dwell times. Compared to the previous HMM treatment (63), which used a more detailed dynamical model, vbTPM does not require curated training data. Instead, it learns the number of states directly from the data along with all other model parameters in a statistically principled way, using a variational Bayes treatment of HMMs (30–35). The number of states, corresponding to, for example, distinct DNA–protein conformations, is often a key quantity of interest, and the possibility to extract it directly from the data will be especially useful for poorly characterized and complex systems (for example, TPM data with three rather than two operators present, as in the wild-type *lac* operon (60)). Also in contrast with previous methods, vbTPM handles common experimental artifacts gracefully, by classifying them in separate states that can easily be filtered out based on their unphysical parameters. Finally, we demonstrate further improved resolution from an ability to pool information from large heterogeneous data sets, using an EB approach (34,35). Combined, these represent significant improvements over previous analysis methods, which we expect to be useful for a wide range of TPM applications. Our code, implemented in a mixture of Matlab and C, is freely available as open-source software.

Our analysis of LacI-mediated loop formation in DNA constructs with loop lengths from 100 to 109 bp is consistent with previous results (16), in the sense that we resolve three states that cluster according to the emission parameters of the model, *K* and *B*, and which we denote the unlooped state (U), middle looped state (M) and bottom looped state (B). Our EB analysis further demonstrates that when the M and B looped states occur in a single trajectory, they can directly interconvert without passing through an unlooped state. This strongly indicates that these M and B states share a DNA-binding topology but differ in LacI conformation, because a change of DNA topology would presumably require an unlooped intermediate, as different DNA topologies require the unbinding and re-binding of at least one LacI DNA-binding domain from the DNA. Our findings of direct interconversions between the M and B states are consistent with previous results on longer (138 bp (13) and 285 bp (14)) loops, which were attributed to transitions between a V-shaped and an extended LacI conformation.

Interestingly, at many loop lengths we can distinguish two kinds of trajectories, those that contain both an M and a B state (which can interconvert), and those that exhibit only one of the two looped states (Figure 5). Which of the looped states (B or M) a two-state trajectory exhibits is the same for essentially all two-state trajectories at a given loop length, but whether this state is the M or B state varies with loop length. As discussed in the Results section and in Section S6, for most constructs we observe significantly more two-state trajectories than we would expect from the null hypothesis that this ‘2+3’ pattern reflects insufficient equilibration of simple three-state kinetics. Although we cannot conclusively rule out the null hypothesis, we find the evidence for two different subpopulations sufficiently compelling to propose an alternative hypothesis, namely the existence of three different underlying loop structures. Taking the 2+3 pattern together with the indication that the single loop state changes with operator phasing (Figure 5), we argue that this pattern reflects the existence of two loop structures that can interconvert directly via a conformational change in LacI, and one structure that cannot interconvert directly to any other looped state, but has the same TPM signature as one of the interconverting states. Interconversion between the two- and three-state regimes is slow compared to our typical trajectory lengths (Figure 6), which is the reason we can distinguish them.

We note that a mixture of two- and three-state trajectories was also seen in a 138-bp construct with directly interconverting looped states, flanked by two Oid operators (13). For a 285 bp loop flanked by two O1 operators, only trajectories with two looped states were reported (14). Closer analysis of these data might be interesting in light of our observations.

**Figure 8.**
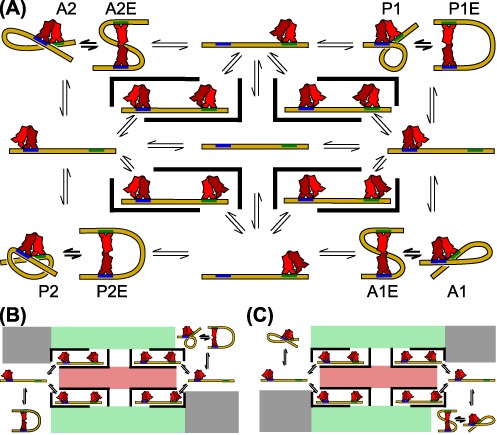
(**A**) Loop structures arranged by LacI binding directions on the operators Oid (blue) and O1 (green). These binding directions determine the loop topology, which, in keeping with the conventions in the literature, we have labeled as A1, A2, P1 and P2. Transitions between loops of different topologies (corners) are only possible via unlooped neighbor states, indicated by double arrows. However, transitions between loops that share binding topologies (e.g. between A1 and A1E, P1 and P1E, etc.) can occur directly, without passing through an unlooped conformation, as we have demonstrated in this work, and are indicated by shorter and thicker double arrows. Singly occupied states can also interconvert via the unoccupied (center) or doubly occupied states, which are here surrounded by thick black bars to indicate forbidden transitions—for example, a doubly occupied state must transition to a singly occupied state before a loop can form. Note that extended LacI conformations may also exist in the unlooped states (20,21), but for reasons of clarity, we have only drawn V-shaped LacI conformations in these cases, to highlight the different binding orientations. (**B**) and (**C**) show two hypothetical divisions of the state space in (A) into two slowly interconverting topology ‘islands’ separated by energetically unfavorable states (grayed out), low probability states (pink) and kinetically rare states (green); see the text for details. In order for these divisions to generate the observed 2+3-state patterns of Figure 5, one of the state ‘islands’ must support only one looped state, while the other must support two looped states that can interconvert with one another. Panels (B and C) illustrate two possible ways to realize such behavior, in which the direct $B\ \rightleftharpoons M$ interconversions are pictured as corresponding to transitions between V-shaped and extended LacI conformations. The two different divisions shown in (B) and (C) might represent in-phase versus out-of-phase operators, which differ in which observed looped state (M or B) is present in two-state trajectories (see Figure 5). For example, under the somewhat simplistic assumption here that the B state we observe by TPM always corresponds to an extended conformation, and the M state to a V-shaped conformation, then panel (B) would represent a hypothetical scenario for out-of-phase operators, which have two B states (one that interconverts with an M state, and one that does not); and (C) would represent in-phase operators, which have two M states (an interconverting one and one that does not interconvert). Since the phasing of the operators determines the amount of twist in the loop, it is plausible that the most energetically favorable loop topologies would change with operator phasing (6–8).

Unraveling the structural basis for this behavior will require further experimental, theoretical and computational efforts beyond the scope of this paper, but it is interesting to speculate about possible underlying molecular mechanisms. We propose as a starting point the scheme outlined in Figure 8. Figure 8A shows various potential loop structures arranged by binding topology (i.e. binding direction on the operators), with loop topology groups separated by unlooped intermediates. Both V-shaped and extended conformations are shown for each group of loop topologies and are depicted as able to interconvert (thicker, shorter double arrows), though it is not clear that all topologies are energetically feasible at the loop lengths we study here, nor that all loop topologies can convert between an extended and V-shaped conformation. Loop formation and breakdown occur via transitions to neighboring unlooped intermediates, as indicated by the thinner, longer double arrows. Singly occupied unlooped states can also interconvert via doubly occupied intermediates.

How could this state space be split into two slowly interconverting subsets as our results suggest? First, we note that for the operators used here, the statistical mechanics analysis from our previous work implies that the no-LacI-bound state (center in Figure 8A) is essentially unpopulated at 100 pM LacI (16), so we have eliminated it as a possible state in our system, as indicated by the pink boxes in Figure 8B and C. Second, we suppose, as shown by gray boxes in Figure 8B and C, that all energetically feasible loops are found only in two diagonally opposite loop topology groups, which therefore form isolated state ‘islands’ separated by energetically unfavorable states. Theoretical and computational work consistently finds some loop topologies to be more stable than others (5,6–8), making this supposition tenable. If we further hypothesize that not all extended states can interconvert with their cognate V-shaped topological equivalents (or vice versa), then we would obtain the mixture of two-state and three-state trajectories that we observe in our data. Interconversions between two- and three-state regimes would then be limited by the need to change LacI binding orientation on the strong operator via multiple unlooped intermediates, which we will argue below is sufficiently slow, given the strength of the operators in our constructs, as to be virtually undetected on the timescales we deal with here.

A final consideration for this scheme relates to the possibility of passing from one state ‘island’ to the other by way of a doubly occupied state. That is, it is possible to move from a loop topology ‘corner’ to a singly bound neighbor state, then to a doubly occupied state, then to the diagonally opposite corner via unbinding of the original LacI. The relatively low frequency of state transitions in our data combined with the relative dissociation rates of LacI for the Oid and O1 operators we use here make this pathway unlikely on the timescales of our trajectories. Oid is about four times stronger than O1 (16,56–59), and off-rates for Oid and O1 under experimental conditions similar to ours have been determined to be about 0.12 min^−1^ and 0.3 min^−1^, respectively (13,69) (similar values have recently been measured *in vivo* as well (70)). Looped and doubly occupied states are therefore almost three times more likely to decay by O1 unbinding, and so we speculate that the unlooped states covered by green boxes in Figure 8B and C act as kinetic barriers between the two outer columns. That is, we hypothesize a very slow interconversion between the binding orientation at Oid for a given trajectory, because unbinding from O1 is so much more likely. Moreover, recent work hints at additional types of unlooped states, which might further slow down transitions between different topology groups (12,18). Over long enough timescales, though, we would imagine that a significant number of trajectories would eventually explore both topology ‘islands’ in either Figure 8B or C, by passing through one of the green boxes.

The scheme we propose in Figure 8 illustrates how our results point to new interesting directions for future investigations into LacI-mediated looping. For example, much theoretical work has focused on looping free energies (5,7,8), which are not enough to address the question of allowed interconversions. Another interesting question is the possibility that great rotational flexibility in LacI, of either the DNA-binding domains (24) or the dimers around the tetramerization domain (20,21), might blur the differences between loop topology groups. Finally, a computational investigation of the RMS signal for different looped states shown in Figure 8, including the effect of the bead and nearby coverslip (7), would aid in matching different structural models directly to TPM data.

Regardless of which molecular structures underlie the interconverting and non-interconverting loop states that we observe, it is clear that our novel Bayesian analysis is central to our ability to resolve evidence for more than two coexisting looped states in a single construct with TPM. This is one more looped conformation than previously observed at the single-molecule level (13–18), but is in qualitative agreement with theoretical and computational results (5–8) (see Introduction). Our findings are also consistent with recent ensemble FRET studies with loops formed from a library of synthetic pre-bent DNAs, in which at least three loop structures (a mixture of V-shaped and extended) contributed significantly to the observed looping for at least 5 of the 25 constructs examined (23).

The impact of these different loop structures on the ability of LacI to regulate the genes of the *lac* operon *in vivo* remains to be seen. Theoretical work has shown that several classic features of *in vivo* gene repression data with LacI can be best explained by the presence of more than one loop conformation, and that the presence of multiple looped states generally dampens oscillations in gene regulation as a function of loop length (4). Extending these arguments, the presence of multiple looped states should allow looping under a wider range of conditions, and hence make gene regulation more robust against mechanical perturbations from, for example, changes in supercoiling state or the presence versus absence of architectural proteins. On the other hand, inducer molecules and architectural proteins such as HU have been suggested to also change the relative stability of different loop shapes (4,8–12) which may add an additional level of regulatory potential to the operon.

The above effects could clearly be present and relevant also in more complex regulatory systems of eukaryotic cells. A fuller understanding of the loop structures and interconversion pathways available to the LacI-mediated loops we observe *in vitro*, and how they are influenced by architectural proteins that are known to play a large role in gene regulation *in vivo* (9–11), promises to greatly enhance our understanding of this potential additional layer of gene regulatory information.

## SUPPLEMENTARY DATA

Supplementary Data available at NAR Online include supplementary information, sections S1-S9, supplementary figures S1-S18, and raw position trace data for the E8106 and E8107 constructs.

SUPPLEMENTARY DATA
