# Supplementary material for: Multiple LacI-mediated loops revealed by Bayesian statistics and tethered particle motion
Source: Nucleic Acids Res. 2014 Aug 12;42(16):10265–77. doi: 10.1093/nar/gku563 (PMC4176382; doi:10.1093/nar/gku563)
Supplement: SUPPLEMENTARY DATA [file supp_42_16_10265__index.html]

Multiple LacI-mediated loops revealed by Bayesian statistics and tethered particle motion — SUPPLEMENTARY DATA 

# Multiple LacI-mediated loops revealed by Bayesian statistics and tethered particle motion

## SUPPLEMENTARY DATA

**Files in this Data Supplement:**

- SUPPLEMENTARY DATA
- SUPPLEMENTARY DATA
