# Supplementary material for: Multiple LacI-mediated loops revealed by Bayesian statistics and tethered particle motion
Source: Nucleic Acids Res. 2014 Aug 12;42(16):10265–77. doi: 10.1093/nar/gku563 (PMC4176382; doi:10.1093/nar/gku563)
Supplement: SUPPLEMENTARY DATA [file supp_gku563_nar-00310-m-2014-File001.pdf]

Supporting information: Multiple LacI-mediated loops revealed by Bayesian statistics and tethered particle motion

S. Johnson, J.W. van de Meent, R. Phillips, C.H. Wiggins, and M. Lindén

Contents

S1 vbTPM workflow 1

S2 The emission parameters 1

S3 Choice of priors 2

S4 Performance on synthetic data 3

S5 Effect of short-lived spurious states 6

S6 Equilibration analysis 7

S7 Detecting less rare interconversions 8

S8 Loop-loop interconversions in all constructs 8

S9 Example trajectories 9

S1 vbTPM workflow

The workflow of vbTPM, summarized in Fig. S1, is based on runinput files that contain all analysis parameters, including information about where the TPM data files are located, and where various results should be written to. These files can therefore be used as handles to an ongoing analysis and to intermediate results.

The three main tools for handling the analysis, marked in yellow in Fig. S1, are
VB7_batch_run.m, which manages the VB analysis of raw position traces using the simple HMM model,
VB7_batch_manage.m, a tool to collect the analysis results, and also to clean up and reset intermediate result files in case the analysis is interrupted, and finally
VB7_batch_postprocess.m, a graphical tool to aid the manual state classification and construct factorial models based on this classification.

More advanced analysis beyond this step, including the EB procedure, currently require custom Matlab scripting. Further details are given in the software manual.

S2 The emission parameters

To gain more physical intuition about the parameters K, B that model the bead motion, we derive the corresponding expressions for the standard deviation (or RMS value) and the correlation time, for the case with no hidden states. The bead motion model, Eq. (**2**) in the main text, can be expressed as a stochastic difference equation whose parameters depend on the hidden state,

$$\boldsymbol{x}_t = K_{s_t}\boldsymbol{x}_{t-1} + \boldsymbol{w}_t/(2B_{s_t})^{1/2}, \tag{S1}$$

where $\boldsymbol{w}_t$ are independent vectors of Gaussians with two independent components and unit variance,

$$\left\langle w_t^{(i)} w_u^{(j)} \right\rangle = \delta_{t,u}\delta_{i,j}, \quad i,j = x,y. \tag{S2}$$

With no hidden states, this simplifies to

$$\boldsymbol{x}_t = K\boldsymbol{x}_{t-1} + \boldsymbol{w}_t/(2B)^{1/2}, \tag{S3}$$

and to compute the corresponding RMS value, we first substitute Eq. (S3) into $\langle \boldsymbol{x}_t^2 \rangle$, to get

$$\left\langle \boldsymbol{x}_t^2 \right\rangle = \left\langle \left(K\boldsymbol{x}_{t-1} + \frac{\boldsymbol{w}_t}{\sqrt{2B}} \right)^2 \right\rangle = K^2 \left\langle \boldsymbol{x}_{t-1}^2 \right\rangle + \frac{\langle \boldsymbol{w}_t^2 \rangle}{2B} + \sqrt{\frac{2}{B}} \left\langle \boldsymbol{w}_t \cdot \boldsymbol{x}_{t-1} \right\rangle. \tag{S4}$$

Now, the equation of motion (S3) means that $\boldsymbol{w}_t$ and $\boldsymbol{x}_{t-1}$ are independent, so that $\langle \boldsymbol{w}_t \cdot \boldsymbol{x}_{t-1} \rangle = 0$, and since $\boldsymbol{x}_t$ is also stationary it follows that $\langle \boldsymbol{x}_t^2 \rangle = \langle \boldsymbol{x}_{t-1}^2 \rangle = RMS^2$. Finally, noting

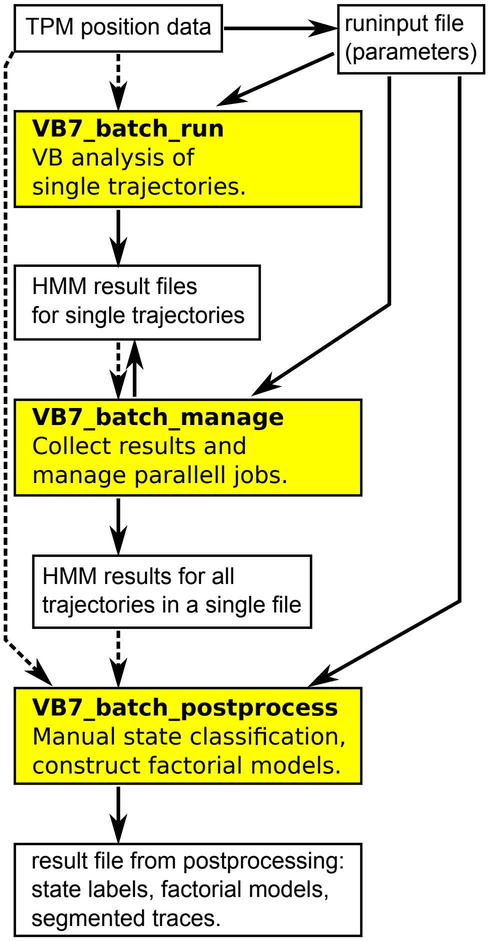

Figure S1: Work flow for TPM analysis using vbTPM. The yellow boxes indicate the three main tools of the vbTPM toolbox and their functions, as described in the text. Solid lines indicate that a file is written by another file, or passed as argument to it. Dashed lines indicate flow of information handled internally by reference to the runinput file.

that $\left\langle \boldsymbol{w}_t^2\right\rangle = \left\langle (w_t^{(x)})^2\right\rangle + \left\langle (w_t^{(y)})^2\right\rangle = 2$, the equation for $\left\langle \boldsymbol{x}_t^2\right\rangle$ simplifies to

$$\left\langle \boldsymbol{x}_t^2\right\rangle = K^2\left\langle \boldsymbol{x}_t^2\right\rangle + 1/B, \tag{S5}$$

which leads to the expression for the RMS value of Eq. (**3**),

$$RMS = \sqrt{\left\langle \boldsymbol{x}_t^2\right\rangle} = \left(B(1-K^2)\right)^{-1/2}. \tag{S6}$$

To derive the correlation time, we similarly start with the equation of motion (S3) to compute $\langle \boldsymbol{x}_t \cdot \boldsymbol{x}_{t-1}\rangle$. After applying the same type of arguments, we get

$$\begin{aligned}\langle \boldsymbol{x}_t \cdot \boldsymbol{x}_{t-1}\rangle &= \left\langle \left(K\boldsymbol{x}_{t-1} + \frac{\boldsymbol{w}_t}{\sqrt{2B}}\right)\cdot \boldsymbol{x}_{t-1}\right\rangle \\ &= K\left\langle \boldsymbol{x}_{t-1}^2\right\rangle + 0 = K\left\langle \boldsymbol{x}_t^2\right\rangle. \end{aligned}\tag{S7}$$

Repeated application to longer times, and division by $\left\langle \boldsymbol{x}_t^2\right\rangle$, leads to

$$\frac{\langle \boldsymbol{x}_t \cdot \boldsymbol{x}_{t-m}\rangle}{\left\langle \boldsymbol{x}_t^2\right\rangle} = \frac{\langle \boldsymbol{x}_{t+m} \cdot \boldsymbol{x}_t\rangle}{\left\langle \boldsymbol{x}_t^2\right\rangle} = K^{|m|} \equiv e^{-|m|\Delta t/\tau}, \tag{S8}$$

where the last step is just the definition of the correlation time τ in terms of the sampling time Δt. This is indeed the correlation time given in Eq. (**3**).

S3 Choice of priors

We would like to choose uninformative prior distributions in order to minimize statistical bias. This is unproblematic for the emission parameters K, B, since the amount of data in all states is large enough to overwhelm any prior influence. As derived in the software manual[1], prior distributions for K, B are given by

$$p(\boldsymbol{K}, \boldsymbol{B}|N) = \prod_{j=1}^{N} \frac{B_j^{\tilde{n}_j}}{W_j} e^{-B_j\left(\tilde{v}_j(K_j-\tilde{\mu}_j)^2+\tilde{c}_j\right)}, \tag{S9}$$

$$W_j = \frac{\tilde{c}^{-(\tilde{n}_j+\frac{1}{2})}\Gamma(\tilde{n}_j+\frac{1}{2})}{\sqrt{\tilde{v}_j/\pi}}, \tag{S10}$$

with the range $B_j \geq 0, -\infty < K_j < \infty$. Throughout this work, we use

$$\tilde{\mu}_j = 0.6, \qquad \tilde{n}_j = 1, \tag{S11}$$

$$\tilde{v}_j = 5.56\ \mathrm{nm}^2, \qquad \tilde{c}_j = 30000\ \mathrm{nm}^2, \tag{S12}$$

which corresponds to

$$\left\langle K_j\right\rangle = 0.6, \qquad \left\langle B_j\right\rangle = 5\times 10^{-5}\ \mathrm{nm}^{-2}, \tag{S13}$$

$$\mathrm{std}(K_j) = 0.3, \qquad \mathrm{std}(B_j) = 141.4\times 10^{-5}\ \mathrm{nm}^{-2}. \tag{S14}$$

The prior for the initial state probabilities are Dirichlet distributed, $p(\boldsymbol{\pi}|N) = \mathrm{Dir}(\boldsymbol{\pi}|\tilde{\boldsymbol{w}}^{(\boldsymbol{\pi})})$, and these variables are unproblematic for the opposite reason: the long length of the trajectories makes the initial state relatively unimportant to describe the data. We use a constant prior of strength 5, i.e.,

$$\tilde{w}_j^{(\boldsymbol{\pi})} = 5/N, \tag{S15}$$

where N is the number of hidden states.

The transition probabilities need more care, because the potentially low number of transitions per trajectory makes the prior relatively more influential. The prior for the transition matrix **A** are independent Dirichlet distributions for each row, parameterized by a pseudo-count matrix $\tilde{w}_{ij}^{(\mathbf{A})}$. Following Ref. [1], we parameterize this prior in terms of an expected mean lifetime and an overall number of pseudo-counts (prior strength) for each hidden state. In particular, we define a transition *rate* matrix Q with mean lifetime t_D,

$$Q_{ij} = \frac{1}{t_D}\left(-\delta_{ij} + \frac{1-\delta_{ij}}{N-1}\right), \tag{S16}$$

[1] See vbtpm.sourceforge.net for the latest version.

and then construct the prior based on the transition probability propagator per unit timestep,

$$\tilde{w}_{ij}^{(\mathbf{A})} = \frac{t_A f_{sample}}{n_{downsample}} e^{\Delta t Q}. \tag{S17}$$

Here, t_A is the prior strength; both t_A and t_D are specified in time units to be invariant under a change of sampling frequency. Further, the timestep is given by $\Delta t = n_{downsample}/f_{sample}$, where f_{sample} is the sampling frequency (30 Hz in our case), and $n_{downsample}$ is the downsampling factor (we use 3).

Numerical experiments in Ref. [1] show that choosing the strength too low compared to the mean lifetime produces a bias towards sparse transition matrices. This is not desirable in our case, and we therefore use $t_D = 1$ s, and $t_A = 5$ s throughout this work.

S4 Performance on synthetic data

Here, we test the abilities of vbTPM to resolve close-lying states in synthetic data, and compare it to the RMS histogram method. We also verify that model parameters are recovered correctly, and that these results are insensitive to the factor three downsampling that we use for analysis on real data.

Our test model, depicted in Fig. S2A, has one unlooped (U) and two looped (M and B) states, and the difficulty of resolving states M and B can be tuned by decreasing either their RMS difference ΔRMS or their average life-time τ_L (the lifetime of the aggregated state B+M is fixed at 30 s, same as the unlooped state). For each parameter setting, we generated and analyzed ten 45 minute trajectories.

Fig. S2B-C shows a comparison of temporal resolution, using ΔRMS=40 nm and varying τ_L. Resolving states using histograms means resolving peaks, and three distinct peaks emerge at $\tau_L = 4-8$ s. In contrast, vbTPM resolves the correct number of states already at $\tau_L = 0.5$ s. This order-of-magnitude improvement mainly reflects the detrimental effects of the low-pass filter used in the RMS analysis, and is insensitive to downsampling by a factor of three. The vbTPM limit can instead be compared to the bead correlation time τ, which were set to 0.1, 0.17, and 0.25 s for the B,M and U states in this data.

We also compared vbTPM to the histogram method for resolving states that interconvert slowly ($\tau_L = 30$ s) with varying degrees of separation in RMS. The result is shown in Fig. S3, and indicates that vbTPM does not significantly outperform the histogram method in this case.

To summarize, we mapped out the resolution of vbTPM in the range 0.0625 s $\leq \tau_L \leq$ 30 s, 5 nm $\leq \Delta RMS \leq$ 40 nm. The results, in Fig. S4, show a nonlinear relation between the spatial and temporal resolution.

Next, we verify that model parameters are also well reproduced and insensitive to downsampling in this situation. Fig. S5 shows the RMS values for the most likely models fitted to the test data set of Fig. S2. The looped state of the two-state models display an average of the two looped states

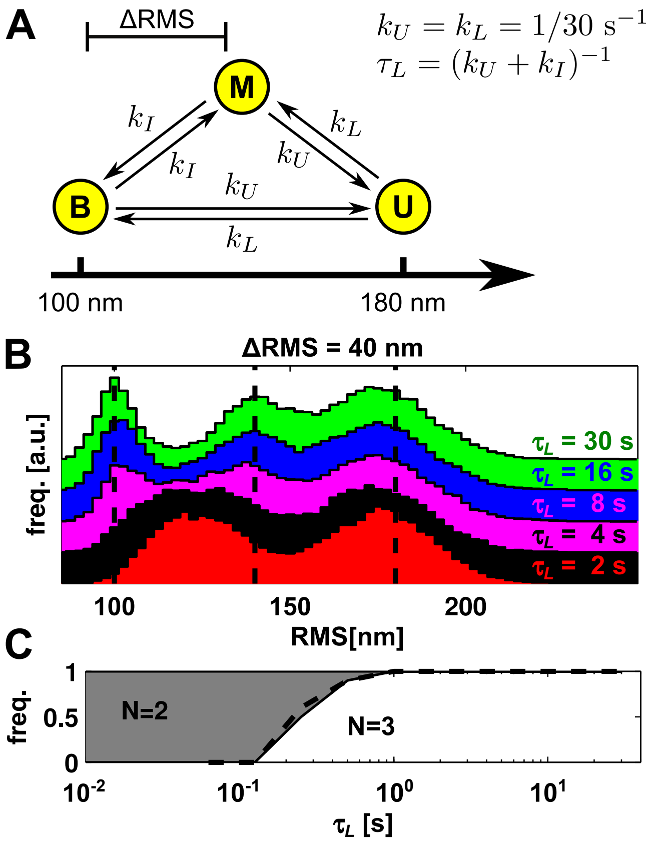

Figure S2: Temporal resolution with vbTPM and RMS histograms. (A) Model for synthetic data, with the difficulty determined by the RMS-separation ΔRMS and mean life time τ_L of the two interconverting states M and B. (B) Aggregated RMS histograms from ten 45-min trajectories with ΔRMS=40 nm and varying τ_L. The M and B states are blurred to a single peak at low τ_L, but for $\tau_L \gtrsim 8$ s, all three states can be resolved. Vertical lines show the true RMS values. (C) Fraction of trajectories in which the HMM algorithm resolved 2 (gray) or 3 (white) states. All three states are resolvable at $\tau_L \geq 0.5$ s, significantly better than the histogram method. The dashed line shows the result without downsampling, an insignificant improvement. The filter width used in (B) was optimized by eye to $\sigma_G = 3$ s.

in the data when those states interconvert too quickly to be resolved. The three-state models generally reproduce the input parameters with a slight downward bias that is more noticeable at high RMS values. We believe that this is an effect of the drift-correction filter we applied to the data. Note that the results with and without downsampling are almost indistinguishable.

The mean lifetimes (Fig. S6) show similar trends of good fit and almost no difference with and without downsampling. Two-state models that do not resolve the two looped states learn their aggregated mean lifetime, which is indeed 30 s in the true model. The tendency to overestimate the short lifetimes can be rationalized by noting that short sojourns are more difficult to resolve, and therefore do not contribute as

much to the estimated parameter values.

Individual transition probabilities (elements A_{ij}) are presented in Fig. S7. Here there is a clear difference with and without downsampling, since the latter estimates transition probability per timestep, while the former per three timesteps. Low transition probabilities suffer significant fluctuations due to small number statistics, while the higher transition probabilities are well reproduced.

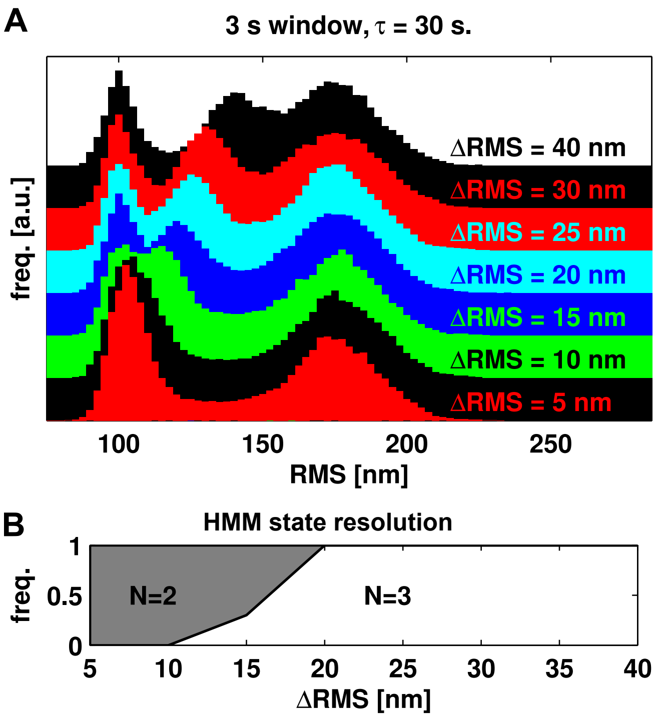

Figure S3: Resolving three states with varying ΔRMS and looped mean life-time τ = 30 s. (A) Aggregated histograms for ten 45 min-trajectories, filtered with σ_G = 3 s. (B) Fraction of detected two- (gray) and three-state (white) models with vbTPM applied to the same ten trajectories one by one.

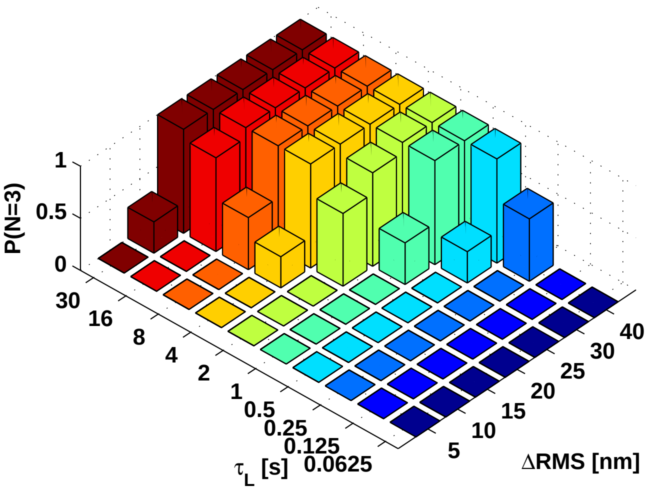

Figure S4: Resolution map of vbTPM shown as the fraction of correctly identified 3-state models at different $(\Delta RMS, \tau_L)$-pairs. Ten 45 min-trajectories were simulated at each parameter set, and 3-fold downsampling was used for the analysis.

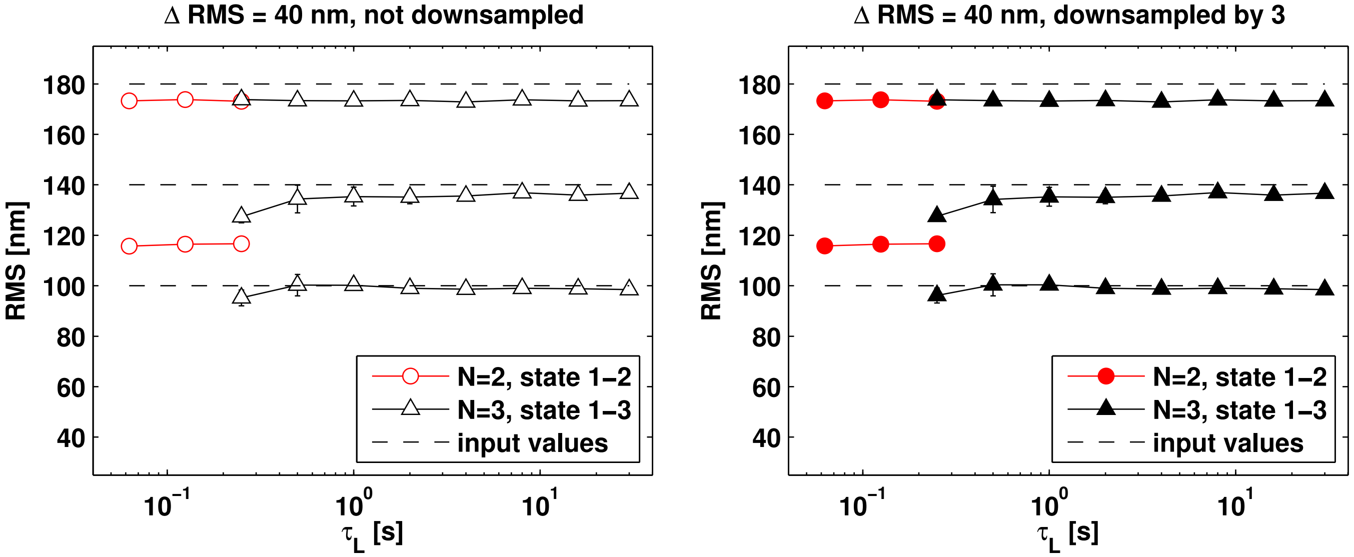

Figure S5: RMS values for the best fit models (symbols) to the data set in Fig. S2, compared to simulated parameters (dashed). Posterior mean value ± std. (an estimate of the parameter uncertainty) for two- and three-state models shown separately, according to which model size got the best score for each trajectory. Most error bars are smaller than the symbols. Analysis without (left) and with (right) downsampling give almost identical results.

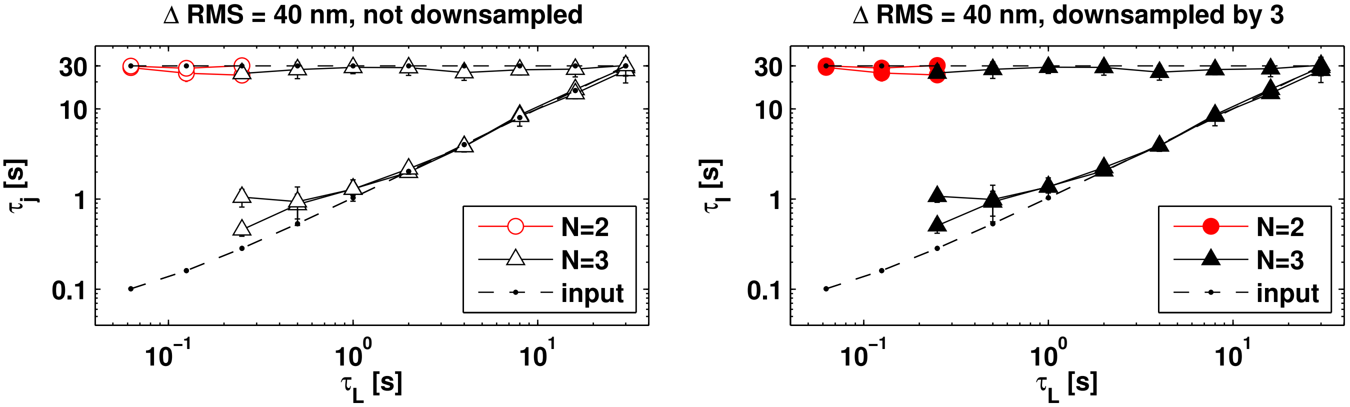

Figure S6: Mean lifetimes, presented as in Fig. S5. The true model (dashed) has one state with mean lifetime 30 s (U), and two states (M and B) with shorter lifetimes. The lower dashed line is not straight because τ_L is defined as a rate in a continuous time model, while lifetimes (true and fitted) are defined in a discrete-time setting using the transition probability matrix A_{ij}, which makes a difference for short lifetimes. The average lifetime of the short-lived states together is always 30 s however, which explains why the two-state models that do not resolve these two states have both lifetimes around 30 s.

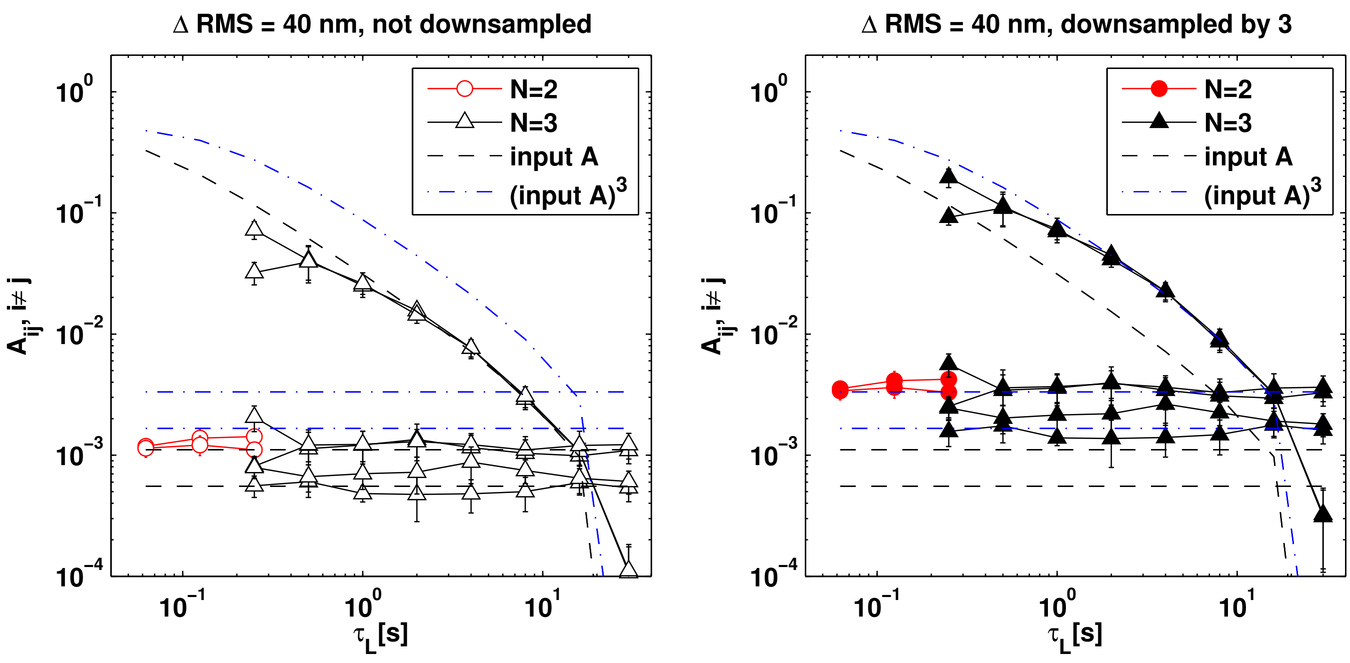

Figure S7: Transition probabilities (non-diagonal elements of A_{ij}), presented as in Fig. S5. Due to symmetries of the underlying kinetic model it only contains three distinct transition probabilities. The difference with and without downsampling is due to the fact that the downsampled model effectively estimates transition probabilities per three timesteps, given by $\mathbf{A}^3$ (blue dash-dotted lines), instead of the single-step probabilities (black dashed lines) used to produce the data. Relative to these different targets, however, the analyses with and without downsampling give very similar results.

S5 Effect of short-lived spurious states

vbTPM is able to detect many short-lived spurious states that cannot be detected in RMS trajectories, and one might wonder if the presence of these states poses a problem for earlier results where they were not detected [2]. To test this, we compute some properties of our E8 constructs subjected to our standard screening process [2] (which does not detect short-lived artifacts), and compare them to a population where the trajectories are subjected to additional screening, namely, where trajectories with the most frequent short-lived spurious events are removed. The differences turn out to be small.

For this additional screening, we looked at the average frequency of transitions from genuine to spurious states and the fraction of time spent in spurious states. As shown in Fig. S8, the distributions of these properties for the E8x and TAx trajectories have distributions that are fairly broad. For this comparison, we set thresholds of at most 6 spurious transitions per minute and 5% spurious occupancy (dashed lines in Fig. S8A,B), which removed about 30% of all trajectories (although the fraction varied significantly between different constructs).

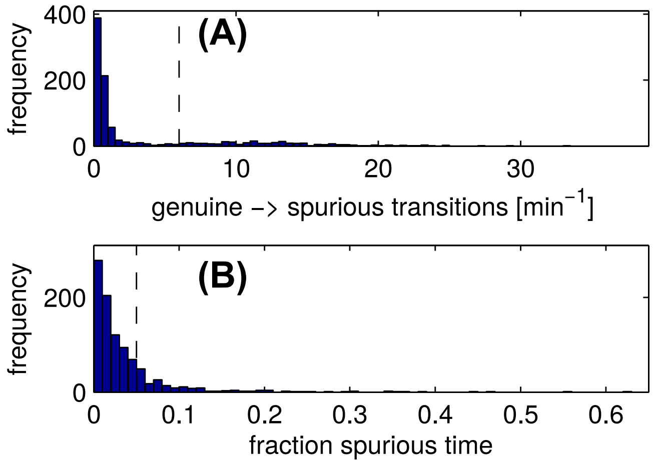

Figure S8: Distribution of short-lived spurious states in trajectories from all (E8x and TAx) constructs. (A) Average frequency of transitions from a genuine to a spurious state. (B) Fraction of time spent in a spurious state.

Figure S9 shows average state occupancies, mean dwell times, and average rates of loop-loop interconversions computed from models converged with the EB algorithm on all trajectories in our E8x constructs (assuming simple three-state kinetics, and should thus be interpreted with care). Solid lines show results for trajectories passing the standard screening, while dashed lines represent the results after the additional screening to remove trajectories with many short spurious events. As seen in Fig. S9, the presence or absence of these “most spurious” trajectories generally have a small effect on the analyzed average properties.

S6 Equilibration analysis

As discussed in the main text, analysis of both E8x and TAx experiments shows that a significant number of trajectories populate only one of two looped states, along with the unlooped state, whereas others populate all three states. One possible explanation for the apparent existence of 2-state and 3-state populations is that we are simply observing equilibration effects, and that every trajectory would eventually populate all three states, provided a bead is observed over a sufficiently long measurement interval. In order to test this null hypothesis—that is, the hypothesis that all trajectories observed for a particular construct are actually drawn from a single, three-state population, and some of them end up only exploring one of the two looped states due to the finite observation time—we have generated datasets consisting of simulated state trajectories, drawn from an underlying 3-state population, and compared the number of 2-state and 3-state trajectories in the simulated data to those found in the analysis of the experimental data.

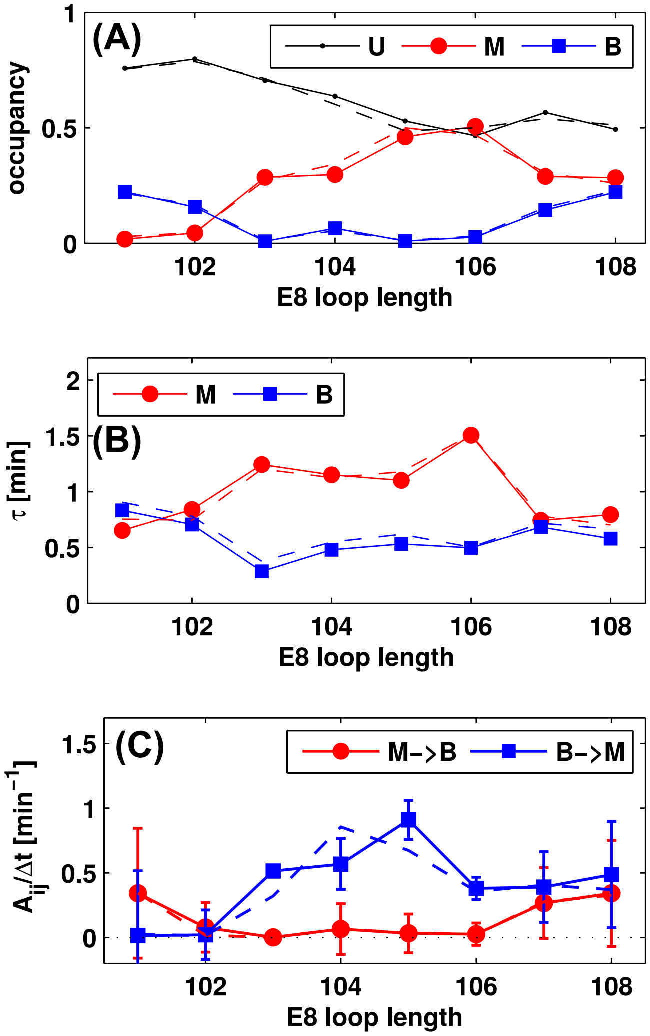

Figure S9: Comparison of (A) state occupancy, (B) mean dwell times, and (C) average transition probabilities between looped states for all E8 trajectories that passed our standard screening (solid) and the additional thresholds defined in this section (dashed). Error bars in (C) are standard deviations. Note that the occupancy values in (A) are not directly comparable to those in our earlier analysis [2], since the effect of trajectories without looping activity was not corrected for in this plot.

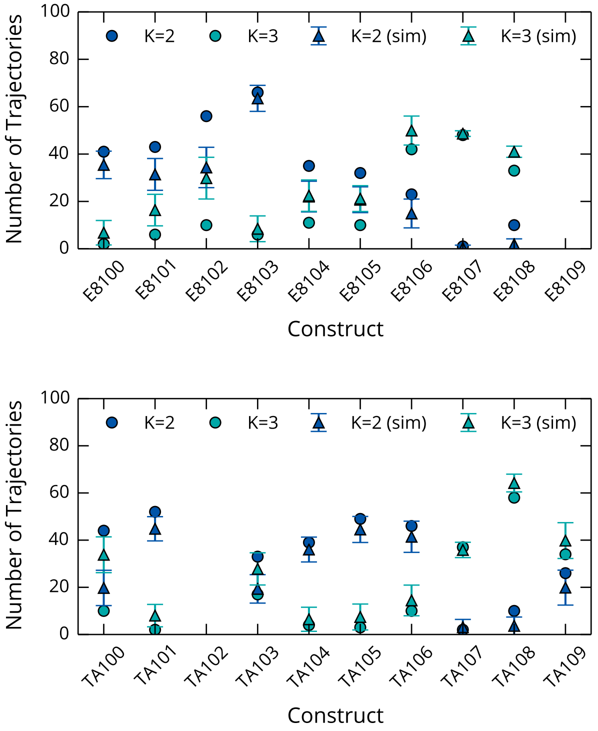

Figure S10: Comparison of the number of 2-state (blue) and 3-state (cyan) trajectories obtained in vbTPM analysis of experimental data (circles) to those in simulated datasets (triangles), for E8x (top) and TAx (bottom) constructs. Error bars mark two standard deviations over 100 simulated datasets. Data for the TA102 and E8109 constructs are missing, because the VB results for those constructs showed too large variability for manual classification.

The procedure for this analysis is as follows. We first perform vbTPM analysis of the experimental data, and then use the EB analysis to estimate a distribution $p(A \mid \alpha)$ over the transition rates and a distribution $p(\pi \mid \rho)$ over the initial state probabilities. (As shown in Fig. S14-S18 below, the EB analysis tends to give more accurate state assignments than the VB analysis, since it uses information from multiple trajectories at once. It also describes the variability between individual beads.) Note that this EB analysis implicitly assumes all trajectories belong to a single 3-state population, though not all trajectories are required to populate each of the 3 states. For each trajectory $n = 1 \ldots N$ in the experiment, we now simulate a trajectory $s_{n,t}$ with a number of time points T_n that is identical to that of the n-th trajectory in the experimental data. To do so, we first sample $A_n \sim p(\cdot \mid \alpha)$ and $\pi_n \sim p(\cdot \mid \rho)$.

We then sample $s_{n,1} \sim p(\cdot \mid \pi_n)$ and $s_{n,t} \sim p(\cdot \mid A_{s_{n,t-1}})$ for $t = 2 \ldots T_n$. We repeat this procedure 100 times, using new values A_n and π_n on each sweep.

Figure S10 shows the number of 2-state and 3-state trajectories obtained through an EB analysis of real data as compared to the corresponding numbers in simulated datasets. In this analysis we define a trajectory as having 3 states when $\sum_t E[s_{n,t,k}] > 5$ for all 3 states k. In other words, a trajectory must assign at least 5 time points to each state in order to be classified as having 3 states. This threshold was empirically chosen to exclude instances where a brief transition to a spurious state may be misinterpreted as a transition to an actual state. However, we verified that analysis results were not qualitatively different when this threshold was lowered to 1 time point for each state. Note that the EB analysis can sometimes find a third genuine state that the VB algorithm missed, as shown in Fig. S16, and thus TA105 does show a few three-state trajectories.

Analysis of the E8x trajectories shows a significantly lower number of 3-state trajectories than in equivalent simulated data. The TAx constructs show a similar, if less pronounced, trend; we believe that this is due to the poor statistics for these constructs, in which the 2+3 pattern is less extreme (that is, fewer TAx constructs have a robust mixture of 2- and 3-state populations, compared to the E8x constructs). Note that for the TAx constructs that do have a significant number of both 2- and 3-state trajectories (*e.g.*, TA100, TA103, TA106, TA109), the trend follows that of the E8x constructs, with more 2-state trajectories observed experimentally than in simulated data. Note also that the error bars on simulated counts show an interval of two standard deviations (95% confidence), which represents a very conservative estimate of the uncertainty. In other words, under the null hypothesis where all trajectories are described by a single 3-state model, we would expect to see a significantly higher number of 3-state trajectories than we actually observe experimentally, suggesting that the 2-state trajectories and the 3-state trajectories in our data are not drawn from the same underlying population. These results lead us to hypothesize that we are observing three looped states, not two, as detailed in the main text.

S7 Detecting less rare interconversions

Fig. 7A shows that the EB algorithm clearly undercounts the number of $B \leftrightharpoons M$ interconversions in the synthetic data based on the E8106 trajectories, where such transitions are very rare. This downward bias is significantly smaller for the synthetic based on E8107 parameters where these transitions are less rare. To see if this trend continues with increasing number of events, we generated and analyzed synthetic data based on E8107 parameters, but with transition rates doubled. This increases the number of events without making dwell times too short. Fig. S11 shows the true and estimated counts for all types of transitions in this data set. Both methods work well on $U \leftrightharpoons B$ interconversions, which have the

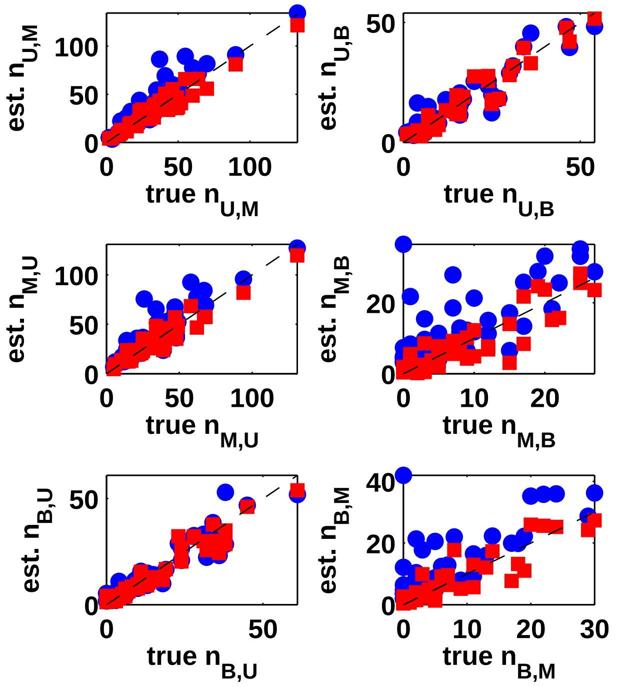

Figure S11: Counting the number of interconversion in synthetic data based on the E8107 parameters with all rates doubled to increase the number of events. VB and EB results are shown in blue and red respectively.

largest RMS difference, but the VB method shows a clear bias on the less well-separated $U \leftrightharpoons M$ and $B \leftrightharpoons M$ interconversions, with greater bias in the latter case, where there are fewer transitions. The EB method appears unbiased in all cases, indicating that the tendency of EB to undercount transitions in the synthetic E8106 data is indeed an effect of rare transitions rather than a systematic downward bias.

S8 Loop-loop interconversions in all constructs

Having established in Fig. 7 that the EB algorithm can reliably detect direct loop-loop interconversions, we present the corresponding analysis for our other constructs. For every construct, we ran EB analysis on all trajectories identified as three-state by the VB algorithm and manual classification, and counted the posterior expectation of the number of BM-interconversions. However, TA105 had no three-state trajectories in this analysis (Fig. 5), so for this construct we instead counted BM-transitions in three-state trajectories based on the EB analysis done for Fig. S10, an analysis that includes all trajectories. The results are shown in Figs. S12-S13.

The EB analysis detects loop-loop interconversions in all constructs. However, since the total number of three-state

trajectories tend to decrease with decreasing loop length, the evidence is most convincing for the longer constructs.

S9 Example trajectories

In Figs. S14-S18, we provide a few examples of analyzed trajectories from the E8106 construct. Each example shows the RMS trace (black), the sequence of most likely hidden states from the VB analysis ("HMM", yellow), the sequence of most likely genuine states from the corresponding factorial model (magenta), and the sequence of most likely states from the empirical Bayes (EB) algorithm, converged with three genuine states on all two- and three-state trajectories (cyan). In some cases, we also show short sections of drift-corrected position traces ($x(t), y(t)$ in blue and red), where the segmentation indicated the presence of short-lived spurious states.

References

[1] Fredrik Persson, Martin Lindén, Cecilia Unoson, and Johan Elf. Extracting intracellular diffusive states and transition rates from single-molecule tracking data. *Nat. Meth.*, 10(3):265–269, 2013. doi: 10.1038/nmeth.2367.

[2] Stephanie Johnson, Martin Lindén, and Rob Phillips. Sequence dependence of transcription factor-mediated DNA looping. *Nucleic Acids Res.*, 40(16):7728–7738, 2012. doi: 10.1093/nar/gks473.

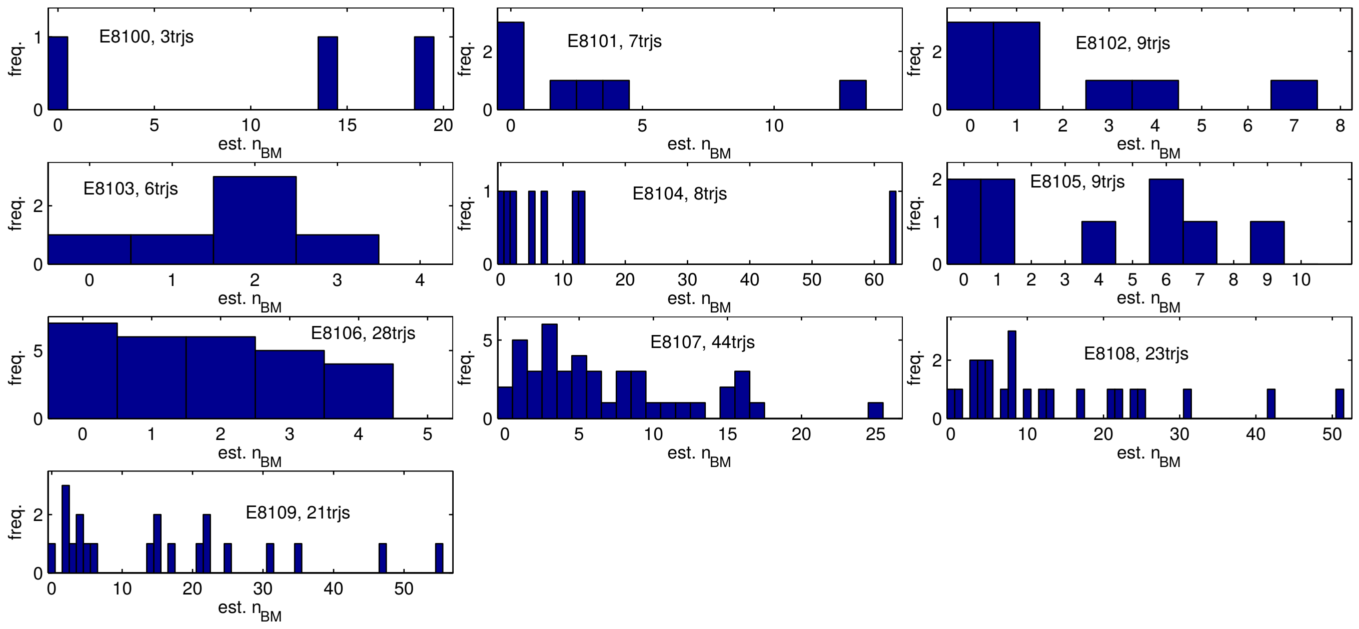

Figure S12: Number of loop-loop interconversions from an EB analysis of three-state trajectories of the E8 constructs, analogous to Fig. 7E,F.

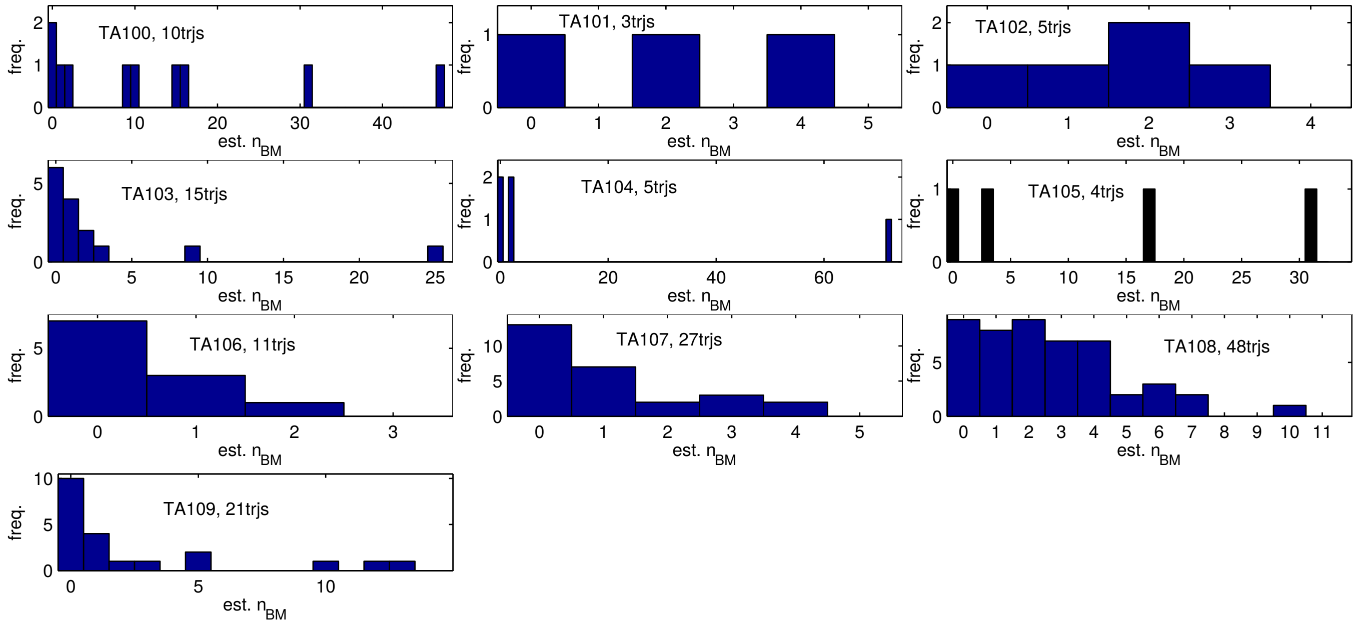

Figure S13: Number of loop-loop interconversions from an EB analysis of three-state trajectories of the TA constructs analogous to Fig. 7E,F. For TA105, which lacks 3-state trajectories in the VB analysis, we use three-state trajectories from the EB analysis of Fig. S10.

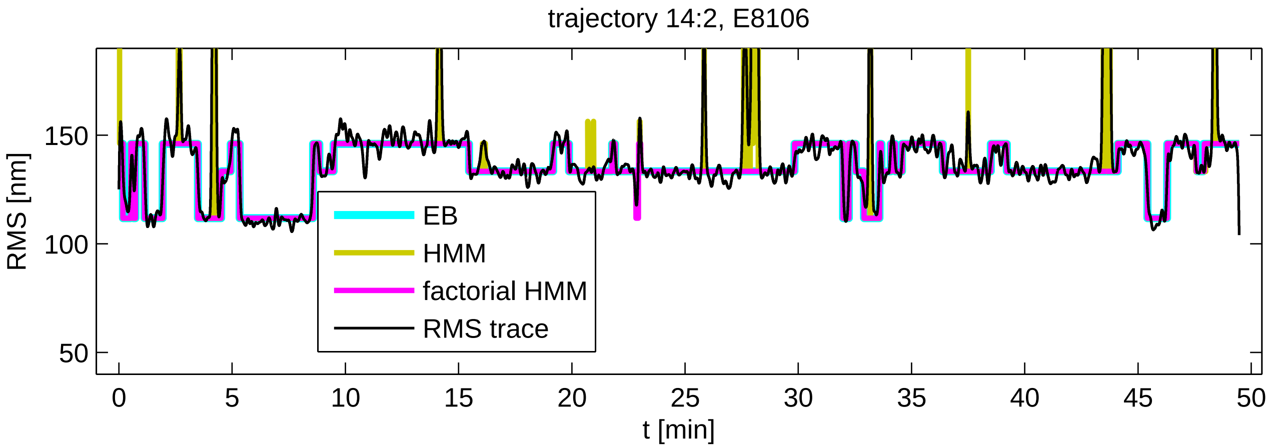

Figure S14: An example of a long, three-state trajectory.

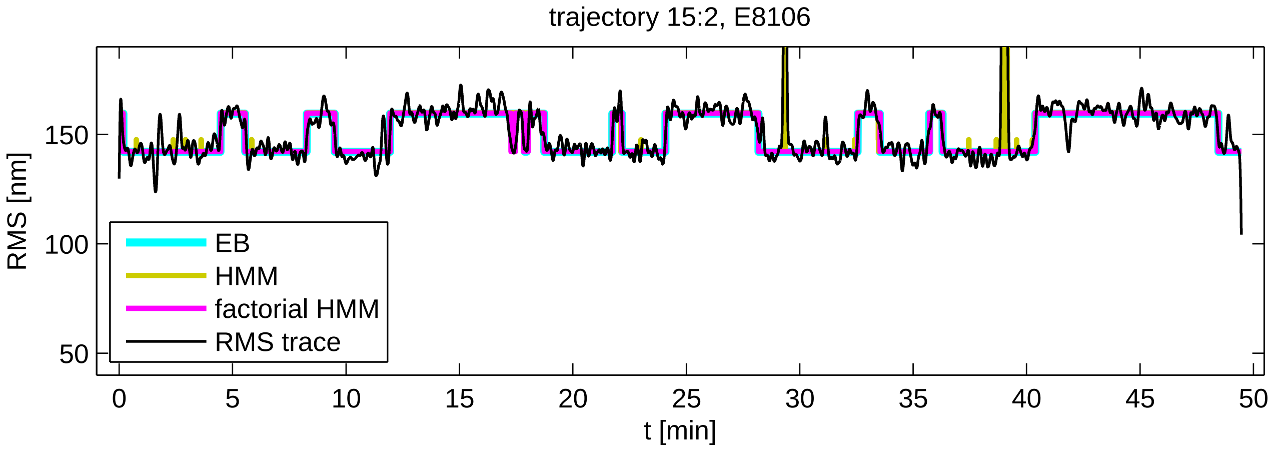

Figure S15: An example of a two-state trajectory of equal length to that of Fig. S14. Note that there are several short, ambiguous excursions of the RMS trace (for example, to a value well below that of the looped state around 2 minutes, and to a value similar to the looped state around 42 minutes) that would be difficult to objectively classify by hand, highlighting one of the advantages of the vbTPM approach. The third state is left unoccupied by the EB algorithm as well, further confirming the 2-state classification.

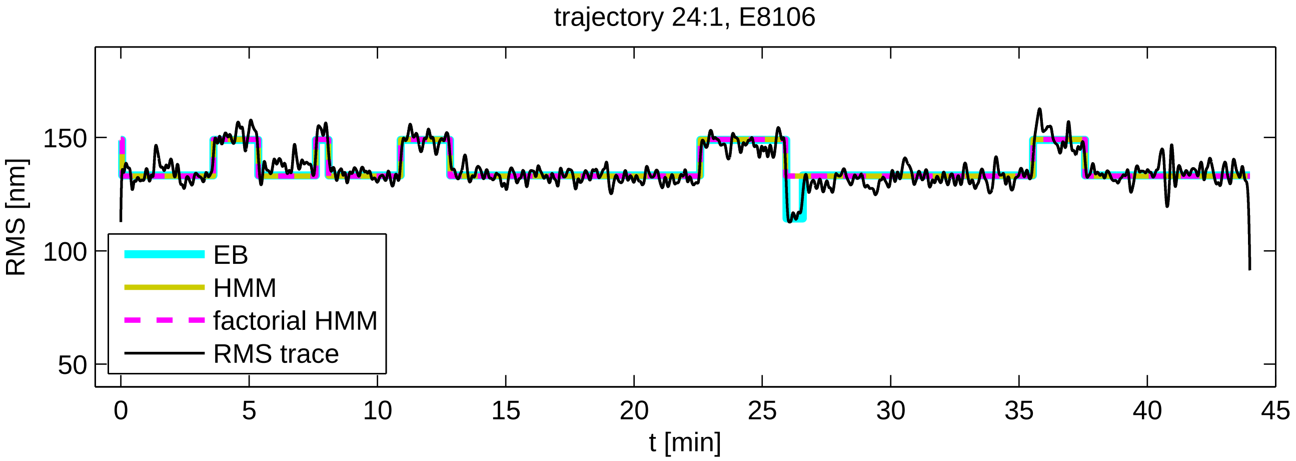

Figure S16: A misclassification of a three-state trajectory as a two-state trajectory by the VB algorithm. The missed third state is only visited briefly, around 26 minutes, but recovered by the EB algorithm. Note that here, there were no spurious states, so the HMM and factorial HMM overlap completely.

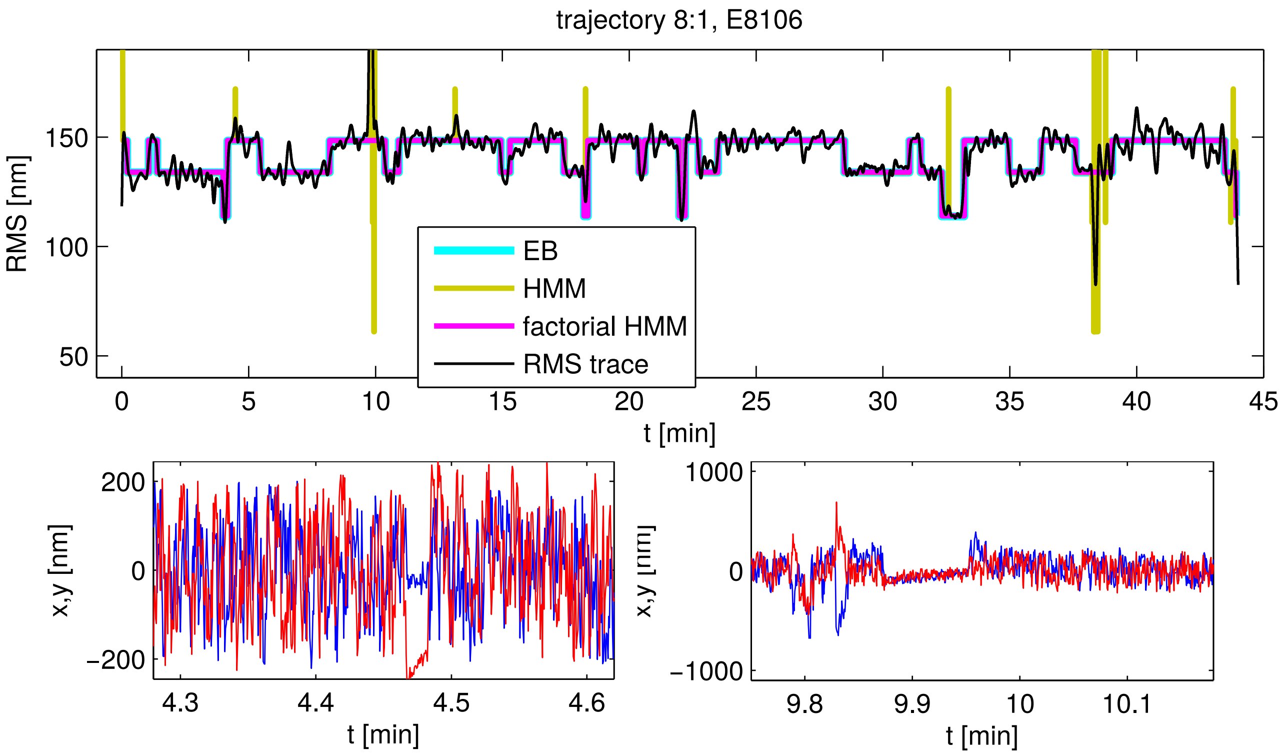

Figure S17: An example of a three-state trajectory with a significant number of spurious states, which the factorial HMM successfully ignores. The $x(t), y(t)$ positions of some of these spurious events are shown in the panels below the main trace, and are probably caused by the bead transiently sticking to the surface. The slow drift towards the origin during these events are caused by the drift-correction filter.

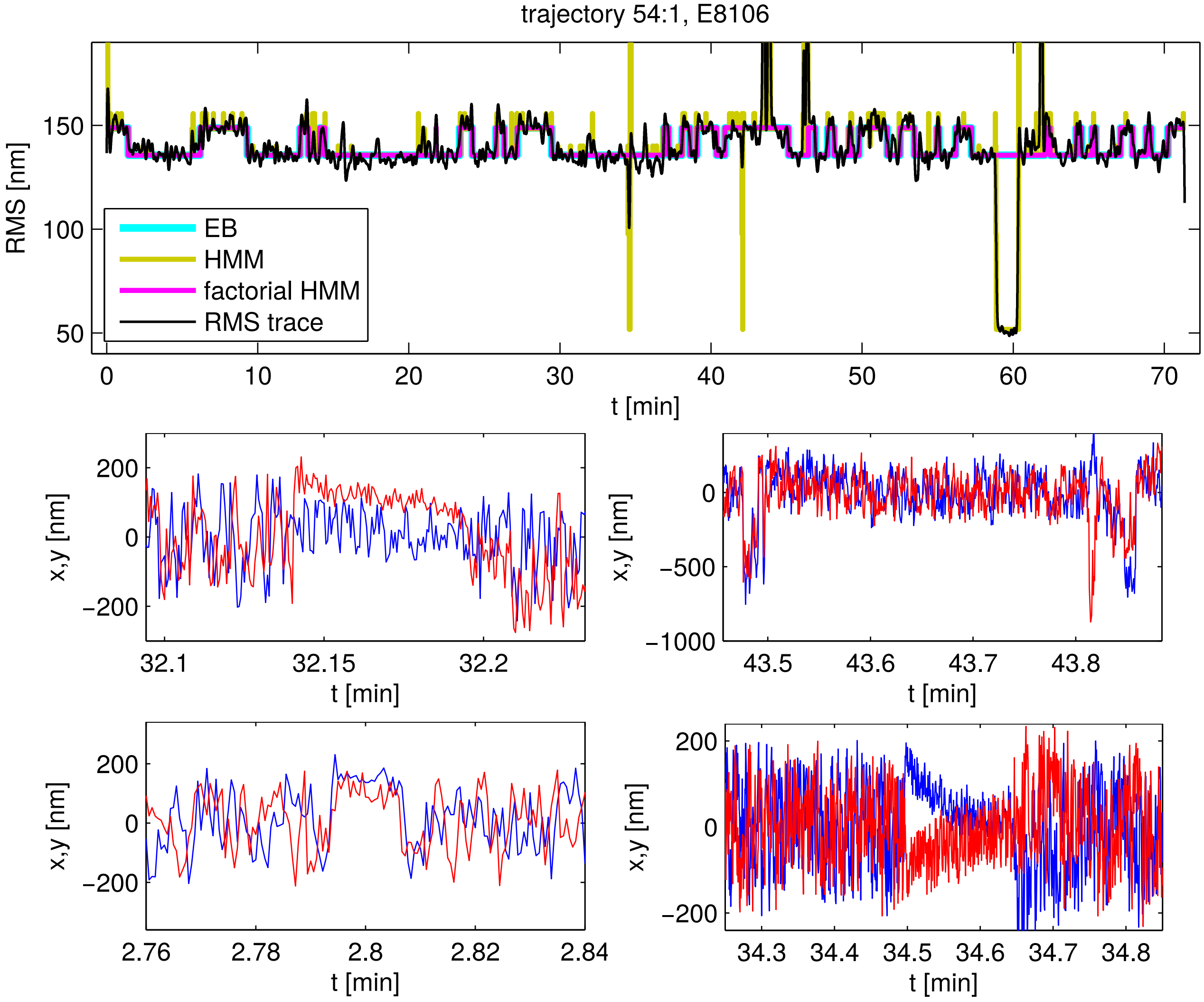

Figure S18: An example of a two-state trajectory with a significant number of spurious states, which the factorial HMM successfully ignores.
